# Fatty Acid Metabolism in Health and Cancer: From Fundamental Mechanisms to Therapeutic Application

**DOI:** 10.1002/mco2.70749

**Published:** 2026-05-18

**Authors:** Na Hang, Runkang Zhao, Fan Zhang, Dandan Guo, Qing Li, Zhijun Shen, Ruiqing Gao, Chenyu Gao, Zhao Xie, Sentao Fu, Peng Luo, Bufu Tang, Ling Wang

**Affiliations:** ^1^ Department of Oncology First Affiliated Hosp Dalian Med Univ Dalian China; ^2^ School of Stomatology Dalian Medical University Dalian China; ^3^ Department of Stomatology Shanghai East Hospital Tongji University School of Medicine Shanghai China; ^4^ Department of Stomatology Jiangxi Provincial People's Hospital Nanchang Jiangxi Province China; ^5^ Department of Oncology Zhujiang Hospital, Southern Medical University Guangzhou China; ^6^ Department of Interventional Radiology Zhongshan hospital, Shanghai Institute of Medical Imaging, Shanghai Institution of Medical Imaging, Shanghai, National Clinical Research Center of Interventional Medicine, Fudan University Shanghai China

**Keywords:** fatty acid metabolism, inflammatory microenvironment, metabolic reprogramming, targeted therapy, tumor microenvironment

## Abstract

Fatty acid metabolism (FAM) plays a vital role in maintaining health by supporting energy production, cellular structure, and signaling processes. However, once this tightly regulated network becomes disrupted, it is increasingly recognized as being linked to the onset and progression of numerous chronic diseases, with cancer being one of the most prominent and clearly defined examples. In addition to the field of oncology, alterations in FAM can also lead to immune dysfunction, inflammatory responses, and metabolic disorders, including diabetes, cardiovascular disease, and neurodegenerative diseases. We examine how different cell types adapt their metabolic behavior within inflamed and tumor‐rich environments, often leveraging FAM to support survival or suppress immune activity. By highlighting recent discoveries in metabolic regulation, intercellular communication, and disease‐specific lipid signatures, we identify new opportunities for therapeutic intervention. These include targeted drugs, gene therapies, nanomedicine platforms, and dietary strategies aimed at restoring metabolic balance. We also discuss the emerging role of fatty acid‐related biomarkers in advancing precision oncology and broader applications in personalized medicine. Together, these insights underscore the centrality of FAM in human health and disease, with particular emphasis on its growing promise as a therapeutic target in cancer and beyond.

## Graphical abstract

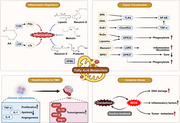

## 1 Introduction

Fatty acid metabolism (FAM) represents a fundamental and dynamic biological network that extends well beyond its traditional role as an energy reservoir. It is essential for maintaining both cellular and systemic homeostasis, serving as a vital source of energy substrates, key structural components of membranes, and a diverse array of bioactive signaling molecules [1]. The precise regulation of fatty acid (FA) synthesis, modification, and oxidation is crucial for several physiological processes, including energy balance, immune function, and neuronal activity [2, 3, 4]. This intricate metabolic system exhibits remarkable plasticity, enabling organisms to adapt to varying nutritional and energetic demands [5].

However, dysregulation of this finely tuned network is a common pathogenic thread that runs through a spectrum of major human diseases. In metabolic disorders like Type 2 diabetes and nonalcoholic fatty liver disease (NAFLD), disruptions in FA flux, characterized by excessive lipolysis, increased uptake, or heightened de novo synthesis, play a significant role in the development of insulin resistance, hepatic steatosis, and lipotoxicity [6, 7, 8]. In atherosclerosis, reprogrammed FAM in vascular endothelial cells, macrophages, and smooth muscle cells fuels chronic inflammation and plaque formation [9, 10]. Similarly, in the tumor microenvironment (TME), aberrant activation of FAM represents a key metabolic adaptation that supports rapid cancer cell proliferation, provides necessary biosynthetic precursors, and actively shapes an immunosuppressive niche [11, 12]. Notably, lipid metabolic reprogramming sustains the functions of immunosuppressive regulatory T cells (Tregs) and tumor‐associated macrophages (TAMs), thereby contributing to resistance against immunotherapy [13, 14]. Furthermore, disturbances in lipid homeostasis are increasingly implicated in neurodegenerative conditions such as Alzheimer's disease, linking peripheral and central nervous system metabolism to neuroinflammation and associated pathology [15].

The complexity of FAM is further amplified by the functional specificity of its components. Different FA species, which are characterized by chain length, degree of saturation, and double‐bond isomerism, exert distinct and sometimes opposing biological effects. For instance, specific polyunsaturated FA (PUFA) isomers have been associated with either protective or detrimental outcomes in chronic heart failure and metabolic diseases [16, 17, 18]. This granularity emphasizes that dysregulation often involves not just a quantitative change in lipid abundance but also a qualitative shift in lipid composition and metabolism [18, 19]. Recent advances in multiomics approaches, including genomics and metabolomics, have begun to elucidate the genetic architecture and molecular mechanisms that govern circulating FA levels, providing new insights into their roles in disease etiology [16, 20, 21].

This review aims to provide a comprehensive synthesis of the dual role of FAM in health and diseases such as cancer. We will first delineate its fundamental mechanisms and vital functions in maintaining physiological homeostasis. Subsequently, we will explore its pathogenic dysregulation across a continuum of diseases, including metabolic disorders, cardiovascular disease, cancer, and neurodegeneration, with particular emphasis on inflammatory and TMEs. Finally, we will discuss the translational potential of targeting FAM, highlighting existing pharmacological strategies, novel therapeutic targets, and future directions for leveraging this metabolic network to enhance human health. This expanded scope underscores the central role of FAM as a critical integrator of metabolic and signaling pathways, whose balanced state is essential for health and whose dysregulation presents a common target for therapeutic innovation.

## 2 Processes in FAM

FAM constitutes a sophisticated physiological process, comprising uptake, synthesis, storage, and oxidation [22]. Exogenous FAs enter the cytoplasm mainly through CD36, FA‐binding protein (FABP), or LDLR [23]. De novo FA synthesis initiates with glucose uptake via GLUT1, followed by its conversion to pyruvate. Subsequently, pyruvate translocates into the mitochondria where it undergoes conversion to citrate. ATP–citrate lyase (ACLY) catalyzes the cleavage of citrate into acetyl‐CoA and oxaloacetate [24]. Acetyl‐CoA carboxylase (ACC) mediates the carboxylation of acetyl‐CoA to malonyl‐CoA, which subsequently undergoes sequential condensation reactions via FA synthase (FASN) to produce palmitic acid [25]. The resultant palmitic acid subsequently undergoes further modification through the action of elongases and desaturases. Specifically, desaturation is catalyzed by stearoyl‐CoA desaturase (SCD) or FA desaturases, whereas elongation proceeds via very‐long‐chain FA elongases, generating FAs with diverse chain lengths and unsaturation degrees [26, 27]. The synthesized FAs are either stored in lipid droplets (LDs) or transported into mitochondria for FA oxidation (FAO) through carnitine palmitoyltransferase 1 (CPT1)‐mediated transport [28]. The oxidative process initiates with FA activation to fatty acyl‐CoA, catalyzed by acyl‐CoA synthetase (ACSL). Following activation, fatty acyl‐CoA undergoes CPT1‐mediated transport into the mitochondria for β‐oxidation [29]. During β‐oxidation, FAs are sequentially degraded to acetyl‐CoA, which subsequently enters the tricarboxylic acid cycle, culminating in ATP generation through oxidative phosphorylation (OXPHOS) to support cellular processes [30].

## 3 The Role of FAM in Health

FAM is a fundamental component of physiological homeostasis, transcending its traditional role in energy storage. In healthy conditions, this complex network supports essential cellular functions through three interconnected pillars: acting as the primary fuel for energy production, supplying critical structural components for cellular membranes, and generating a diverse array of bioactive signaling molecules. This section will systematically explore how the coordinated processes of FA synthesis, modification, and oxidation contribute to energy homeostasis, membrane integrity, signal transduction, and immune competence, thereby providing a foundational framework for understanding its dysregulation in disease states.

### 3.1 Energy Homeostasis

FAs serve as primary energy substrates and sophisticated signaling molecules in systemic energy homeostasis. Historically, their evolutionary significance stems from being a portable energy resource that provides 9 kcal per gram—more than double the energy yield from glucose—while requiring less water for storage, thus facilitating mobility and survival in energy‐scarce environments [31]. This historical advantage persists in modern physiology, though its implications have shifted dramatically in contexts of energy abundance.

The hypothalamus functions as the master regulator of energy homeostasis through its nutrient‐sensing neurons that detect peripheral metabolic status. FAs and their metabolites act as critical signaling molecules in these hypothalamic circuits, modulating neuronal activity by altering mitochondrial dynamics, glycosylation, and influencing neurotransmitter release [32, 33, 34]. Emerging research reveals a compelling link between the activation of Ppp1r17 neurons in the dorsomedial hypothalamus and systemic antiaging benefits. This neuronal activation drives sympathetic signaling to enhance physical activity and optimize white adipose tissue function, thereby yielding a notable extension of lifespan in mice models [35]. This central lipid‐sensing mechanism represents a sophisticated system for monitoring energy flux and adjusting metabolic programs accordingly.

When this regulatory system becomes overwhelmed by chronic lipid excess, as occurs in obesity, the resulting lipotoxicity and metabolic dysfunction disrupt normal energy homeostasis, contributing to the pathogenesis of Type 2 diabetes [36, 37]. Elevated circulating free FAs (FFAs) characteristic of insulin‐resistant states not only reflect adipose tissue dysfunction but actively participate in disease progression by inducing oxidative stress, promoting endothelial dysfunction, stimulating inflammatory cytokine release, and directly provoking insulin resistance in peripheral tissues [31, 38, 39, 40]. Interestingly, analysis of neurophysiological mechanisms suggests that a high‐fat diet disrupts the relative concentrations of polyunsaturated (PUFA) and monounsaturated FAs (MUFA) in the hypothalamus. This imbalance leads to circadian rhythm disruption, ultimately impairing the adaptation of circadian rhythms to seasonal photoperiods [41].

In summary, FAs are crucial for maintaining energy homeostasis, acting as both energy sources and signaling molecules. The hypothalamus plays a pivotal role in regulating these processes, with disruptions in lipid metabolism contributing to metabolic disorders such as Type 2 diabetes. Understanding these mechanisms is essential for developing strategies to combat obesity‐related complications and promote healthy aging.

### 3.2 Regulation of Membrane Function

FAs are essential structural components of biological membranes. Their chemical properties, particularly the degree of saturation and chain length, significantly influence membrane fluidity, thickness, and the formation of specialized microdomains, including lipid rafts [42]. In addition to their structural contributions, FAs and their phospholipid derivatives actively modulate the function of integral membrane proteins. By influencing the conformational states and activity of ion channels, transporters, and signaling receptors embedded within the lipid bilayer, these lipids exert direct regulatory control over membrane‐mediated signaling and transport processes [43, 44, 45, 46]. This dynamic interplay is exemplified by the protein‐mediated incorporation of FAs into membranes via transporters like CD36, which facilitates targeted lipid delivery and influences membrane remodeling in response to metabolic cues [47, 48].

Emerging research has illuminated the pivotal role of FAs in orchestrating membrane contact sites between organelles. For instance, ESYT1, ESYT2, and VAPB form a complex at tricontact sites involving LDs, mitochondria, and the endoplasmic reticulum, facilitating FA transfer for β‐oxidation—a process essential for cellular lipid homeostasis [49]. In yeast, a novel tripartite structure termed the “subcellular bearing,” composed of the vacuole, LDs, and the nucleus, has been identified. This structure highlights the central role of LDs in its assembly and demonstrates how it promotes lipophagy to efficiently degrade LDs under nutrient stress [50]. Intriguingly, also in yeast, LD‐localized LDO proteins, which contain an intrinsically disordered region, directly interact with the vacuolar protein Vac8 to form vacuole–LD contact sites (vCLIP). Nutrient stress enhances vCLIP formation and disruption of vCLIP impairs lipophagy and compromises lifespan extension induced by caloric restriction [51].

Given the exceptionally high lipid demand at synapses, which arises from continuous membrane trafficking associated with neurotransmission, it is understandable that local FAM is indispensable for sustaining synaptic function. Recent evidence shows that Synaptotagmin 1 facilitates the coupling of synaptic vesicle exocytosis and endocytosis by promoting the local synthesis of the signaling lipid phosphatidylinositol 4,5‐bisphosphate, thereby maintaining presynaptic membrane homeostasis [52]. Furthermore, FABPs in the brain interact with neuroactive lipids such as EETs and modulate EET‐mediated synaptic gating, thereby regulating the strength of glutamatergic transmission in the hippocampus [53]. Dysregulation of membrane lipid composition is increasingly implicated in neurological disorders, highlighting the therapeutic potential of targeting FAM to restore membrane homeostasis and cellular function [54, 55, 56]. Notably, the influence of FAM in the central nervous system extends beyond synaptic regulation. Various lipids and their derivatives contribute to metabolic reprogramming, nuclear receptor activation, and membrane microdomain remodeling, collectively influencing the proliferation, lineage specification, and maturation of neural stem cells [56].

Collectively, these findings underscore that FAs are not merely passive structural components of membranes but active determinants of membrane architecture and functionality. They serve as key integrators of metabolic and signaling networks across a wide range of physiological contexts.

### 3.3 FAs as Signaling Precursors

FAs are indispensable precursors for a vast array of bioactive signaling molecules that regulate physiology [57]. PUFAs such as omega‐6 arachidonic acid and omega‐3 eicosapentaenoic acid (EPA) and docosahexaenoic acid (DHA), are esterified in membrane phospholipids and released upon enzymatic stimulation to serve as the primary substrates for multiple signaling pathways [58, 59]. These liberated PUFAs are metabolized by cyclooxygenase (COX), lipoxygenase (LOX), and cytochrome P450 enzymes into diverse mediator families, including eicosanoids and specialized proresolving mediators (SPMs), which play pivotal yet opposing roles in immune regulation [60]. Beyond these oxygenated derivatives, FAs are also conjugated to other molecules to form novel signals; for instance, their enzymatic linkage to neurotransmitters creates lipidated neurohormones that relay metabolic status to modulate behavior via nuclear receptors like NHR‐49/PPARα [61]. Crucially, FAs and their derivatives transduce signals by activating specific cell surface and nuclear receptors. They engage G protein‐coupled receptors (GPCRs) to initiate rapid cellular responses and bind to peroxisome proliferator‐activated receptors to directly regulate the transcription of genes governing metabolism and inflammation [62, 63]. Thus, through their enzymatic conversion into potent mediators and their direct receptor interactions, FAs constitute a central signaling network that integrates metabolic and inflammatory homeostasis while its disruption has been believed as a cornerstone of pathophysiology. The following sections will detail the roles of diverse lipid derivatives in inflammation and the TME; accordingly, this overview has been intentionally concise.

### 3.4 Immunoregulation

FAM serves as a fundamental mechanism that regulates immune cell fate, function, and overall immune homeostasis. Immune cells undergo dynamic metabolic reprogramming upon activation, shifting their utilization of FAs between FAO for energy production and de novo lipogenesis (DNL) for membrane biosynthesis and signaling molecules, a process crucial for determining their functional phenotypes [64].

The differentiation and function of CD4+ T cell subsets are particularly sensitive to FAM. The balance between inflammatory T helper 17 (Th17) cells and anti‐inflammatory Tregs is decisively regulated by the metabolic switch between DNL and FAO. Specifically, the differentiation of inducible Treg cells driven by TGF‐β1 signaling depends on a KLHL25–ACLY module that degrades ACLY, a key enzyme for DNL, thereby shifting metabolism toward FAO. Conversely, interleukin (IL)‐6 signaling antagonizes this pathway, stabilizing ACLY to promote DNL and support the proinflammatory Th17 cell differentiation [65]. This intricate metabolic switch ensures appropriate immune responses and prevents autoimmunity. In CD8+ T cells, impaired lipid metabolism contributes to functional exhaustion across different types of cancer. In hepatocellular carcinoma (HCC), enhanced lipid metabolism in tumor cells leads to the accumulation of FFAs in the TME, which promotes exhaustion of CD8+ T cells via CD36‐mediated activation of STAT3 signaling [66]. Similarly, FFAs released by tumor‐associated adipocytes induce lipid peroxidation in CD8+ T cells, disrupting mitochondrial fitness and driving T cell dysfunction [67]. Notably, recent work reveals that γδ T cells in adipose tissue respond to circadian rhythms by rhythmically producing IL‐17, which in turn dynamically regulates DNL in the fat tissue [68]. This discovery effectively illustrates a coherent bidirectional circuit that links FAM with immune regulation.

Beyond T cells, FAs and their metabolites modulate immunity via specific receptors. Short‐chain FAs (SCFAs), such as acetate, propionate, and butyrate produced by gut microbiota, interact with GPCRs (e.g., FFARs) and influence the activity of various immune cells, thereby regulating inflammatory reactions and energy homeostasis [69, 70]. Furthermore, PUFAs give rise to a vast array of bioactive mediators. For instance, the omega‐3 PUFAs serve as precursors for SPMs, which actively promote the resolution of inflammation [71]. Dietary intake of omega‐3 PUFAs, such as α‐linolenic acid from linseed oil, can be converted into antiallergic and anti‐inflammatory metabolites, highlighting the role of specific FAs in fine‐tuning immune responses [72].

The functional states of key immune cells such as macrophages, dendritic cells (DCs), and B cells are critically influenced by lipid metabolic pathways. In lipid‐associated macrophages, the TM4SF19 protein modulates lysosomal acidification and the capacity to clear apoptotic and necrotic adipocytes, thereby influencing obesity‐associated systemic inflammation and insulin sensitivity. Conversely, genetic deletion of TM4SF19 has been shown to improve metabolic function [73]. In DCs, the WTAP–m6A–ALOX15 axis enhances the secretion of arachidonic acid‐derived metabolites, including LTB4, 12‐HETE, and 15‐HETE. These lipid mediators, in turn, promote cutaneous inflammation and disrupt lipid homeostasis through cross‐talk with keratinocytes [74]. Separately, the SCFA valerate reinforces an anti‐inflammatory milieu by augmenting IL‐10 production in B cells [75].

In summary, FAM is indispensable for immune regulation, governing immune cell differentiation, function, and the balance between proinflammatory and anti‐inflammatory states. Understanding these mechanisms provides a metabolic perspective on immune homeostasis and unveils potential therapeutic avenues for immune‐related diseases.

### 3.5 Summary and Perspectives

FAM is essential for health. It serves as a versatile integrator of energy provision, structural integrity, and signal transduction. The interdependence of these roles is significant. The same lipid species can fuel oxidation, form cellular membranes, and initiate signaling cascades. This interdependence highlights a fundamental principle of metabolic economy and specificity.

Future research should focus on deciphering the “fatty acid code” that dictates these functional outcomes. Developing strategies to restore metabolic flexibility across tissues will also be crucial. Ultimately, a comprehensive understanding of this network is vital for unlocking its significant therapeutic potential to maintain physiological homeostasis and address disease.

## 4 The Role of FAM in Multiple Diseases

Building upon the fundamental understanding of FAM in maintaining physiological health, it is now evident that the dysregulation of this intricate network is a pivotal contributor to a broad spectrum of human diseases. Beyond its well‑documented contributions to metabolic syndromes, cardiovascular diseases, and inflammatory disorders, aberrant FAM is particularly prominent and functionally indispensable in cancer development and progression. As highlighted in the preceding sections, reprogrammed FAM supports sustained tumor proliferation, facilitates invasion and metastasis, endows cancer cells with therapy resistance, and remodels the TME in ways that promote malignant progression. Among this vast landscape of diseases, cancer stands out as a paradigm of metabolic dysregulation, where the reprogramming of FAM is not just a bystander effect but a core hallmark that fuels malignancy. Therefore, a comprehensive understanding of FAM in both malignant and nonmalignant diseases will provide critical insights into shared and disease‑specific regulatory mechanisms, laying a rational foundation for the development of targeted therapeutic strategies. Against this background, we herein summarize the multifaceted roles of FAM in multiple diseases, with a special emphasis on its distinctive functions in cancer.

### 4.1 FAM in Inflammation

Inflammation, as a fundamental physiological response, serves a dual purpose in maintaining tissue homeostasis and defending against external insults [76, 77]. However, dysregulation of this process is a central factor in numerous pathological conditions [78, 79, 80]. An increasing body of evidence indicates that metabolic reprogramming, particularly concerning FAs, acts as a crucial regulator that connects initial inflammatory triggers to sustained alterations in the microenvironment [81, 82]. FAM has irreplaceable importance in many aspects of life activities, especially in biological processes such as cell proliferation, apoptosis, differentiation, and immune regulation [83, 84, 85]. In proliferating cells, FAM generates both energy and lipid precursors essential for membrane biosynthesis [84]. During apoptosis, perturbations in FAM, exemplified by ceramide accumulation, directly activate apoptotic signaling cascades [85, 86]. FAM also regulates cell fate determination through modulation of mitochondrial function and cellular energetics [87].

**FIGURE 1:**
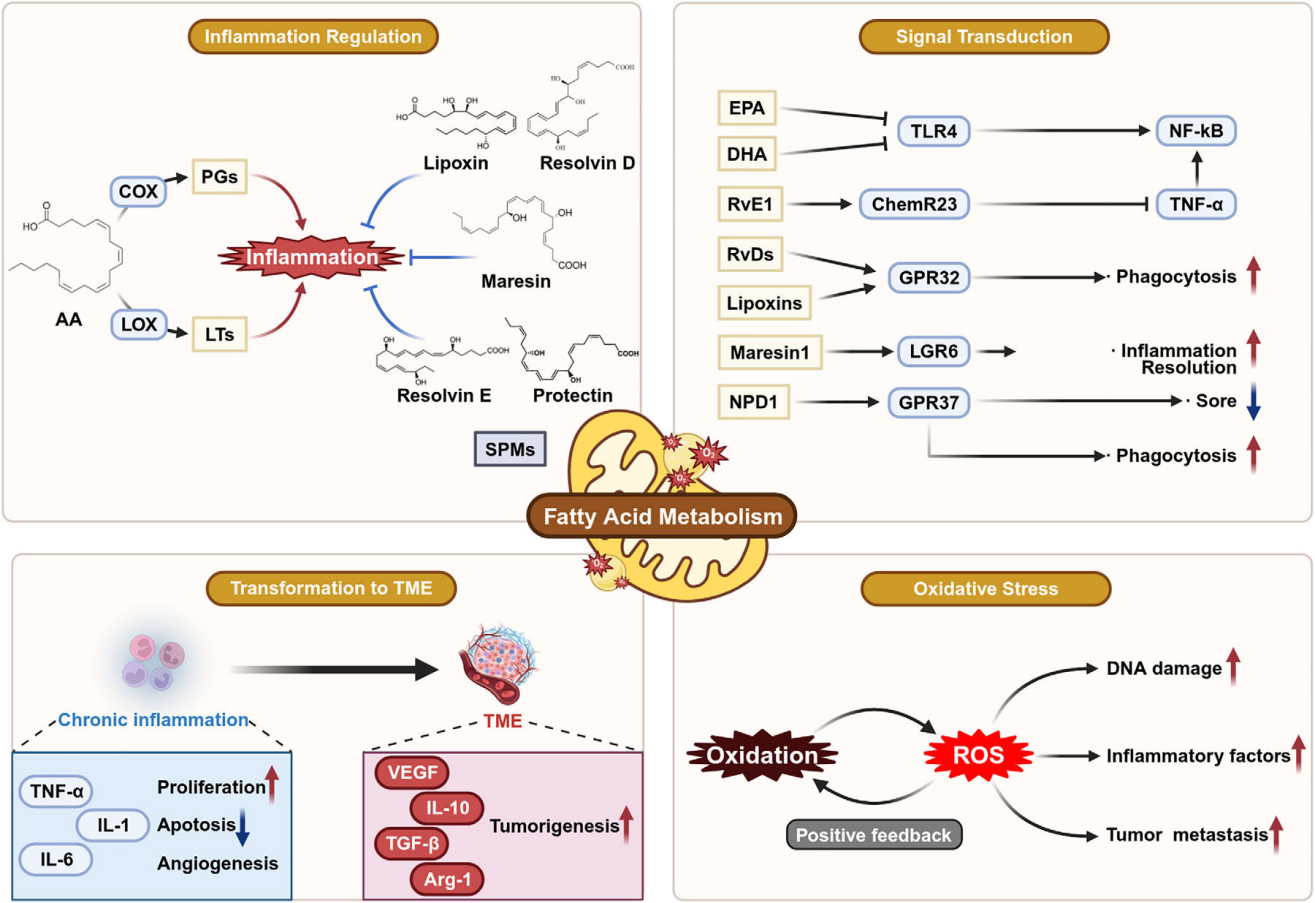
The role of fatty acid metabolism in the TME. The modulation of the TME by fatty acid metabolism primarily occurs through four key pathways: inflammation regulation, signal transduction, translation to TME, and oxidative stress.

Emerging evidence demonstrates that FAM constitutes a critical component in inflammatory microenvironment reprogramming, orchestrating inflammation initiation, progression, and its transition toward the TME [88, 89, 90] (Figure 1). The inflammatory microenvironment is a specific microenvironment formed under inflammatory conditions, composed of various inflammation‐related substances, including proinflammatory enzymes, inflammatory cells, and inflammatory mediators [91]. FAM exhibits pleiotropic effects within this microenvironment, influencing inflammation initiation, progression, and resolution. Dysregulation of FAM during the inflammation‐cancer transition orchestrates microenvironmental remodeling, facilitating malignant transformation [92]. FAM simultaneously supports cancer cell bioenergetics and biosynthesis while promoting inflammation‐cancer transition through regulation of inflammatory mediators and immune responses [93]. Specifically, inflammatory cell‐derived cytokines and chemokines trigger reprogramming of FAM in tumor cells, promoting their proliferative and viability. This metabolic interplay serves as a critical factor in transforming an inflammatory microenvironment into TME [94]. In this framework, FAM not only sustains the bioenergetics and biosynthetic processes of cancer cells but also contributes to the establishment of an immunosuppressive microenvironment.

In the following sections, we will delve deeper into specific aspects of FAM in inflammation, including the production of inflammatory mediators and the balance between proinflammatory and anti‐inflammatory signals, the mechanisms of signal conduction, the role of oxidative stress and the transformation of the inflammatory microenvironment to the TME.

#### 4.1.1 Inflammatory Mediator Production and Proinflammatory and Anti‐Inflammatory Balance

Arachidonic acid, a PUFA, undergoes metabolism through COX and LOX pathways, yielding inflammatory mediators including prostaglandins and leukotrienes [95, 96, 97]. These mediators orchestrate inflammatory cell recruitment, activation, and proliferation, while inducing vasodilation, thus amplifying the inflammatory response [97, 98, 99]. Specific resolution pathways subsequently facilitate the restoration of tissue homeostasis. Inflammatory regression is initiated through the bioconversion of inflammatory mediators, wherein classic prostaglandins and leukotrienes are transformed into resolvins, which function as SPMs with immunomodulatory properties [100]. The SPM family encompasses omega‐6 arachidonic acid‐derived lipoxins and omega‐3 EPA/DHA‐derived metabolites, including resolvin D series (RvDs), resolvin E series (RvEs), protectins, and maresins [100, 101]. These bioactive mediators facilitate inflammatory resolution, attenuate inflammatory cell infiltration, and enhance tissue repair processes [102, 103, 104]. The coordinated actions of these protective lipid mediators govern inflammatory resolution, establishing homeostasis between pro‐ and anti‐inflammatory processes.

#### 4.1.2 Signal Conduction

Omega‐3 FAs (EPA and DHA) inhibit inflammation by suppressing toll‐like receptor 4 activation, blocking downstream signaling cascades to reduce inflammatory signaling, and interfering with NF‐κB transcriptional initiation to decrease the expression of inflammatory genes [105]. Distinct SPM classes mediate inflammatory resolution via specific GPCR binding, thereby modulating cellular signaling pathways and inflammatory cell functions. GPR32 (ALX/FPR2) was first characterized as a resolvin receptor, demonstrating selective high‐affinity binding for RvDs [106]. Lipoxins mediate their anti‐inflammatory effects predominantly via GPR32 binding, enhancing macrophage phagocytic activity and attenuating neutrophil recruitment and infiltration [107]. Arita et al. [108] demonstrated that RvE1, via signaling through the specific receptor ChemR23, inhibits or attenuates tumor necrosis factor (TNF)‐α‐induced NF‐κB activation, thereby mediating its anti‐inflammatory and proinflammatory resolution effects. Chiang et al. [109] confirmed that maresin 1, by activating the LGR6 receptor, stimulates phagocytes to perform key proinflammatory functions in the anti‐inflammatory process. Neuroprotectin D1 (NPD1) is a SPM derived from the omega‐3 PUFA DHA, and it has been shown that GPR37 is a potential receptor for NPD1, which binds to NPD1 and promotes phagocytosis by macrophages while reducing inflammatory pain [110]. Collectively, omega‐3 FAs and their bioactive SPM derivatives orchestrate inflammatory responses and resolution via multiple signaling pathways.

#### 4.1.3 Oxidative Stress

In the inflammatory microenvironment, FAM orchestrates inflammatory responses through multiple mechanisms and exhibits intricate associations with oxidative stress, cellular injury, and disease progression [90, 111]. Reactive oxygen species (ROS), produced during FAM, are key regulatory factors. Excessive ROS induces cellular and DNA damage, triggers lipid peroxidation, and stimulates inflammatory cytokine release, thereby establishing a feed‐forward loop between oxidative stress and inflammation that promotes FAM [112]. This self‐perpetuating cycle critically influences the pathogenesis and progression of chronic inflammatory conditions and associated malignancies. FAO‐derived metabolites modulate inflammatory cytokine expression profiles. Specifically, saturated FAs, exemplified by palmitic acid, activate NF‐κB signaling pathways, thereby upregulating inflammatory cytokine expression in macrophages [90]. In contrast, omega‐3 FAs primarily exert anti‐inflammatory effects by modulating the production of eicosanoids and cytokines [113]. This dual regulatory paradigm underscores the complex biological functions of FAM within the inflammatory microenvironment.

Oxidative stress is an important bridge between chronic inflammation and disease development, and sustained oxidative stress not only activates inflammation‐related signaling pathways, but also leads to DNA damage and genomic instability, which promotes the development of chronic diseases such as cancer [114]. For example, Meira et al. [115] found that chronic inflammation‐induced DNA damage leads to colon cancer development in mice, underscoring the contribution of oxidative stress in tumorigenesis. However, cells are not defenseless against oxidative stress. Activation of the Nrf2 signaling pathway significantly upregulates the expression of antioxidant enzymes, enhances antioxidant capacity, and mitigates cellular damage caused by oxidative stress [111, 116]. This protective mechanism can, to a certain extent, break the vicious cycle of oxidative stress and inflammatory response, protecting cells from further damage.

#### 4.1.4 Transition of the Inflammatory Microenvironment to the TME

Chronic inflammation promotes carcinogenesis through diverse mechanisms, including genomic instability induction, enhancement of cell proliferation and survival, establishment of an immunosuppressive microenvironment, and facilitation of tumor invasion and metastasis. During chronic inflammation, the persistent presence of inflammatory cells and factors causes damage to cellular DNA, leading to gene mutations and promoting tumorigenesis [117]. Inflammatory cells (e.g., macrophages and neutrophils) secrete various cytokines such as TNF‐α and ILs (IL‐1, IL‐6), which promote cell proliferation, inhibit apoptosis, and induce angiogenesis, creating a beneficial microenvironment for tumor progression [117]. Inflammatory cells and mediators establish an immunosuppressive milieu within the TME [118]. Studies have shown that TAMs secrete immunosuppressive factors, such as TGF‐β and arginase, which inhibit antitumor immune responses, facilitate tumor immune evasion, and allow for continued tumor growth and progression [119]. Simultaneously, soluble factors released by tumor cells activate specific signaling pathways, synergizing with TME‐induced metabolic reprogramming to jointly drive TAM self‐renewal and maintain their protumor phenotype [120]. Therefore, targeting these key factors or metabolic nodes holds promise as an anticancer strategy by simultaneously inhibiting TAM self‐renewal and function. Furthermore, inflammatory factors and cells promote tumor cell invasion and metastasis. For example, inflammatory factors such as VEGF induce angiogenesis, providing a blood supply for tumor cell metastasis [121]. Chronic inflammation promotes the accumulation of immunosuppressive cells, including Tregs, myeloid‐derived suppressor cells (MDSCs), and TAMs, further compromising antitumor immunity [119]. Studies have reported that Tregs inhibit the function of effector T cells and reduce the body's immunosurveillance capabilities against tumors by secreting immunosuppressive factors such as IL‐10 and TGF‐β [122]. These findings demonstrate that inflammation plays a pivotal role throughout cancer pathogenesis, from tumor initiation and progression to therapeutic responses. The persistent presence of inflammatory cells and factors, their interactions with tumor cells, the shaping of the TME by inflammatory factors, and the establishment of an immunosuppressive milieu collectively provide important avenues for developing new cancer prevention and treatment strategies. Table 1 delineates the roles and signaling pathways of inflammatory mediators in cancer. A deeper understanding of these mechanisms will help us better target inflammatory pathways, break immunosuppression, and thus bring new hope to cancer treatment.

**TABLE 1:** Inflammatory factors: Functions and signaling in cancer.

Inflammatory factor	Source cells	Signaling pathways	Function	References
TNF‐α	Macrophage, T cell	NF‐κB, MAPK	Promotes cell proliferation, inhibits apoptosis, induces angiogenesis, and promotes tumor cell invasion and metastasis	[123]
IL‐1	Macrophage, DCs	NF‐κB	Promotes inflammatory response, induces tumor cell invasion and metastasis, and promotes angiogenesis	[124]
IL‐6	Macrophage, T cell	STAT3, JAK–STAT	Promotes cell proliferation, inhibits apoptosis, and induces an immunosuppressive microenvironment	[125]
IL‐8	Macrophage, endothelial cells, tumor cell, CAFs	PI3K/AKT, MAPK, NF‐κB	Promotes tumor cell proliferation, angiogenesis, invasion, and metastasis, as well as immune escape	[126]
IL‐10	Tregs, macrophage	JAK–STAT	Suppression of antitumor immune responses, promotion of immune escape, and maintenance of an immunosuppressive microenvironment	[127]
IL‐12	DCs, macrophage, B cell	JAK–STAT	Activation of NK cells, CTLs, and Th1, enhancement of antitumor immune response, inhibition of angiogenesis and immunosuppressive microenvironment	[128]
IL‐17	Th17	NF‐κB, MAPK, IL‐6–STAT3	Promotes inflammatory response, induces angiogenesis, and promotes tumor cell proliferation	[129, 130]
IL‐23	DCs, macrophage	JAK–STAT	Promotes inflammatory response, supports tumor cell survival, and induces an immunosuppressive microenvironment	[131, 132]
TGF‐β	TAMs, Tregs	SMAD	Suppresses immune response, promotes EMT and tumor metastasis	[133, 134]
VEGF	Tumor cell, TAMs	PI3K/AKT, MAPK	Induces angiogenesis, promotes tumor growth and metastasis	[135, 136, 137]

*Abbreviations*: CAFs: cancer‐associated fibroblasts; NK cells: natural killer cells; CTLs: cytotoxic T lymphocytes; Th1: T helper cell 1; EMT: epithelial–mesenchymal transition.

### 4.2 FAM in Cancer

Cancer continues to pose a significant challenge to global health with millions of new cases and deaths annually and projections indicating a substantial increase by 2050, primarily due to its relentless proliferation, ability to metastasize, and frequent development of resistance to therapies [138, 139, 140]. For many years, oncological research has justifiably focused on the cancer cell itself, seeking to uncover intrinsic oncogenic drivers and vulnerabilities. However, the limited success of numerous therapies targeting only tumor cells has highlighted an incomplete understanding of the disease [141]. In recent decades, a paradigm shift has occurred, recognizing that tumor behavior is not solely determined by malignant cells but is significantly influenced by their dynamic interactions with the surrounding stroma [141, 142]. This realization has led to the conceptualization of the TME—a complex ecosystem comprising diverse immune cells, fibroblasts, blood vessels, signaling molecules, and extracellular matrix components [143]. Within this specialized niche, a continuous dialogue between cancer cells and noncancerous elements governs tumorigenesis, progression, and therapeutic responses [144].

**FIGURE 2:**
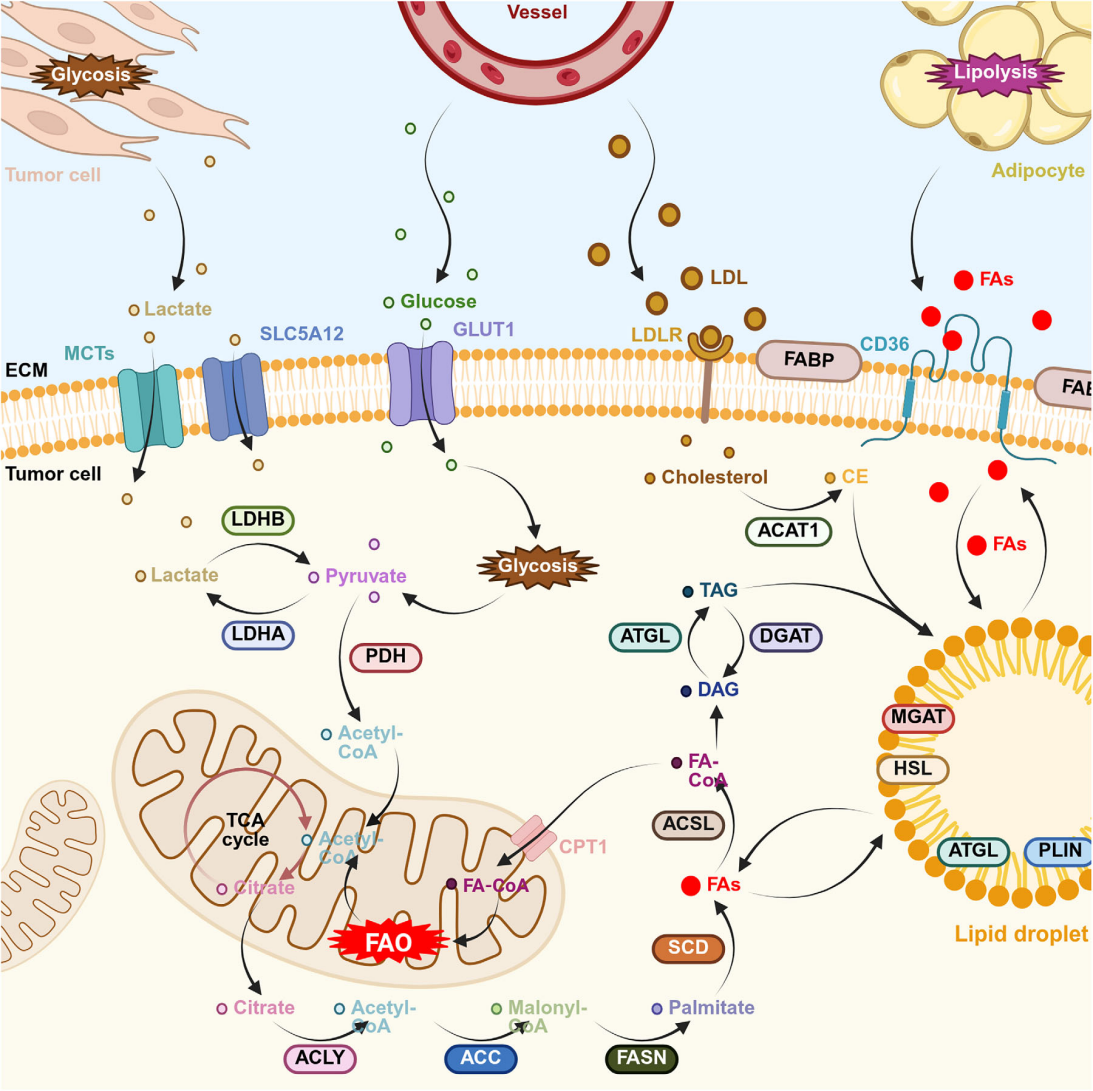
Fatty acid metabolism pathways in tumor cells. Tumor cells acquire fatty acids through two primary mechanisms: (1) direct uptake of exogenous FFAs released from tumor‐associated adipocytes via lipolysis, followed by storage in LDs for utilization; and (2) de novo lipogenesis from glucose and lactate. Lactate, via lactate dehydrogenase, and glucose, through glycolysis, generate pyruvate, which is converted to acetyl‐CoA by pyruvate dehydrogenase and enters the mitochondria. Following the tricarboxylic acid (TCA) cycle, citrate is exported to the cytoplasm, where ACLY reconverts it to acetyl‐CoA. Acetyl‐CoA is then carboxylated to malonyl‐CoA by ACC and subsequently converted to palmitate by FASN. Finally, SCD desaturates palmitate to generate other fatty acids. Interestingly, cytosolic fatty acids can be converted to fatty acyl‐CoAs by acyl‐CoA synthetase long‐chain family member (ACSL). These fatty acyl‐CoAs then have two fates: (1) mitochondrial import via CPT1 for FAO and energy production; or (2) synthesis of diacylglycerol (DAG) through a series of enzymatic reactions, followed by triacylglycerol (TAG) formation via adipose triglyceride lipase (ATGL) and incorporation into LDs. Concurrently, exogenous low‐density lipoproteins (LDLs), via LDL receptor (LDLR), enter the cytoplasm, yielding cholesterol which is esterified to cholesteryl esters (CE) by ACAT1 for LD synthesis.

A critical insight gained from studying the TME is the metabolic interplay and competition for nutrients between tumor and immune cells [145]. Among various metabolic pathways, FAM has emerged as a key regulator of both tumor cell viability and immune function [146, 147, 148] (Figure 2). The reprogramming of this metabolic pathway is a defining characteristic of many cancers, serving to meet the biosynthetic and energetic demands of rapidly proliferating tumor cells while simultaneously shaping an immunosuppressive TME. This metabolic reconfiguration directly impacts on the effectiveness of contemporary immunotherapies and plays a crucial role in defining the immunological landscape of tumors, which are often classified as “hot” or “cold” [148, 149, 150]. Hot tumors are characterized by significant T‐cell infiltration and heightened immune activity, which typically correlates with improved responses to immune checkpoint inhibitors (ICIs). Conversely, cold tumors exhibit a profoundly immunosuppressive TME, characterized by a scarcity of cytotoxic T cells and the presence of immunosuppressive populations such as MDSCs and M2‐type macrophages. This classification holds clinical significance, as patients with cold tumors generally face poorer prognoses and derive limited benefits from immunotherapy [151, 152, 153].

Consequently, the role of FAM in the TME is both multifaceted and essential. The subsequent sections will explore its specific mechanisms: it directly fuels tumor cell growth and proliferation; it serves as a critical mediator of immune regulation, influencing the function of various immune subsets; and it acts as a key driver of therapeutic resistance, presenting substantial challenges to effective treatment.

#### 4.2.1 FAM and Tumor Cell Growth

FAs significantly influence both the composition and function of cell membranes through a variety of ways, including forming the phospholipid bilayer structure of cell membranes, regulating membrane stability and fluidity, and participating in the biosynthesis of cell membranes [154, 155, 156]. These membrane‐associated functions assume heightened significance in tumor cells, which upregulate FA synthesis and uptake while utilizing FAO for energy generation to facilitate microenvironmental adaptation and sustain rapid proliferation [30, 94]. It has been found that acidosis‐induced TGF‐β2 activation in cancer cells of various origins stimulates an increased capacity for FA uptake by cancer cells through PKC‐zeta‐mediated CD36 metastasis, and that cancer cells may directly utilize these FA stores to generate energy, thereby supporting their efficient metastasis and dissemination [157]. This metabolic adaptation confers survival advantages to tumor cells within the hypoxic and nutrient‐limited TME. Therefore, FAM is not only essential for maintaining cell membrane function but also represents a key determinant of tumor cell growth and survival.

Beyond their roles in membrane formation and energy storage, FAs, through their metabolic intermediates, participate in the synthesis of signaling molecules and angiogenesis. For example, the FA metabolite arachidonic acid, via the LOX pathway and subsequent mTOR activation, promotes cell proliferation and neovascularization, significantly impacting breast cancer progression [158]. Neovascularization provides tumors with essential oxygen and nutrients. Arachidonic acid derivatives, such as prostaglandins, function as signaling molecules influencing tumor cell proliferation, migration, and invasion. Prostaglandin E2 (PGE2), in particular, is a significant player in the TME, exhibiting diverse signaling functions. It promotes the invasiveness of epithelial tumors through mechanisms that modulate immune responses, stimulate cancer cell proliferation and migration, and induce angiogenesis [159]. PGE2 elicits subtype‐specific biological responses through differential EP receptor activation: EP1 promotes tumor cell migration/invasion, EP2 drives angiogenesis while suppressing antitumor immunity, and EP4 facilitates metastatic dissemination [160]. Increasing evidence demonstrates that the PGE2/EP signaling pathway is closely associated with the development of lung cancer. Recently, it has been found that PGE2 activates extracellular signal‐regulated kinase 5 through the EP1 receptor, which in turn promotes tumor invasiveness in non‐small cell lung cancer (NSCLC), and therapeutic strategies targeting this signaling axis may be important for controlling lung cancer progression [161]. In conclusion, FAM significantly influences tumor cell growth, which not only provides cells with essential energy and synthetic substances, but also promotes tumor progression through signaling and promoting angiogenesis. These insights establish a rationale for developing anticancer therapies targeting FAM.

#### 4.2.2 FAM and Immune Regulation in the TME

**FIGURE 3:**
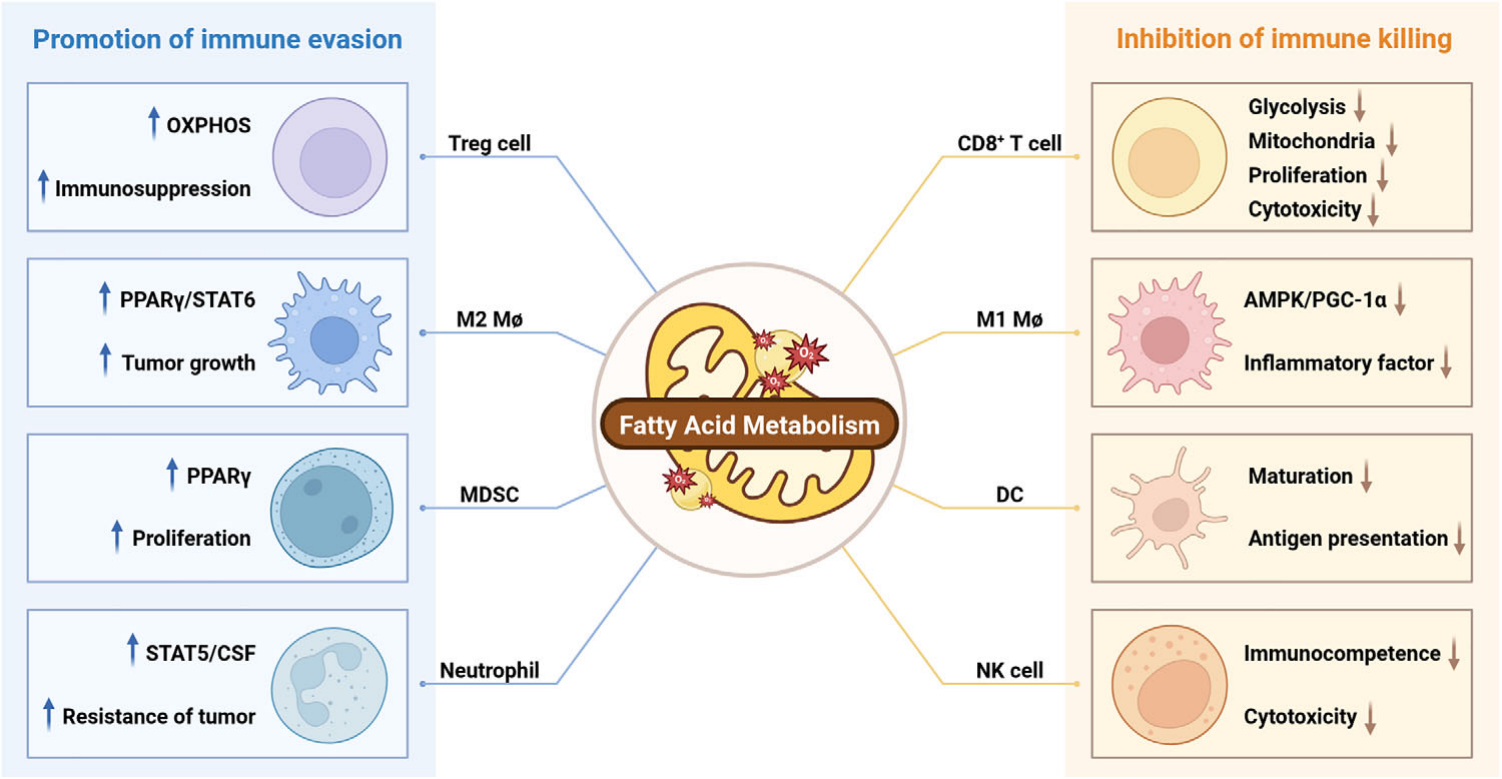
Dual immunomodulatory roles of fatty acid metabolism in the TME. Fatty acid metabolism promotes immune evasion by enhancing the activity and proliferation of immunosuppressive cells. This metabolic adaptation promotes their survival and functional maturation, fostering an immune‐tolerant niche. Conversely, it inhibits immune killing by suppressing the survival and metabolic function of immune effector cells.

Tumor cells and stromal components within the TME collectively shape an immunosuppressive milieu that dysregulates immune cell function. Tumor cells utilize FAM to regulate immune cell function, subsequently facilitating immune escape [162, 163] (Figure 3). The following sections delineate key FA metabolic enzymes that regulate immune cell function in the TME through distinct mechanisms.

CD36, a multifunctional transmembrane glycoprotein, serves as a FA translocase that facilitates the uptake of exogenous FAs [164, 165]. Expression of CD36 is ubiquitously distributed within the TME, spanning tumor cells, stromal cells, and immune cells. Research has shown that increased levels of FAs in TME promote the generation of Tregs, which depend on the uptake of exogenous FAs for their immunosuppressive function. Upregulated CD36 in Tregs enhances mitochondrial metabolism through PPAR‐β signaling, allowing metabolic adaptation to lactate‐abundant TME and promoting tumor immune evasion [166]. Additionally, CD36‐mediated FA uptake in tumor‐infiltrating CD8+ T cells compromises their antitumor effector function, and elevated CD36 expression correlates positively with tumor progression [167]. In summary, these results demonstrate CD36's regulatory effects on TME immune cell functionality and its contribution to tumor immune escape.

FASN, a pivotal enzyme in de novo FA synthesis, critically regulates immune cell activation, differentiation, and function within the TME, shaping immune infiltration and tumor immune evasion [168]. FASN contributes to tumor immune evasion by suppressing proinflammatory immune cells while enhancing the infiltration and function of immunosuppressive M2 macrophages and Tregs within the TME [169]. Studies by Lim et al. [170] established that FASN‐mediated FA biosynthesis is crucial for Tregs functional maturation, a process critical for maintaining immune homeostasis; within the TME, Tregs attenuate antitumor immunity and promote immune evasion through multiple mechanisms, including the secretion of immunosuppressive cytokines (e.g., IL‐10 and TGF‐β) and the competitive consumption of IL‐2 with respect to effector T cells. Recent investigations have further elucidated the dual function of FASN in modulating FA synthesis and restricting T cell immune responses, thereby promoting tumor evasion of immune surveillance and therapeutic resistance [171].

ACC1, as the rate‐limiting enzyme in FAM, significantly impacts the energy utilization of CD8+ T cells in the TME. Research has demonstrated that ACC1 activity in the TME induces LD accumulation in CD8+ T cells, while ACC1 inhibition augments FAO, consequently enhancing the persistence and tumor control of CD8+ tumor‐infiltrating lymphocyte [147]. Moreover, ACC1, through its regulation of FA biosynthesis, exerts a critical influence on CD4+ T cell memory potential. It was shown that gene deletion of ACC1 promotes the formation of CD4+ T memory cells [172]. Th9 cells, a distinct subset of CD4+ T cells, predominantly secrete IL‐9 and demonstrate diverse functions, encompassing antitumor immunity and allergic inflammation. Recent studies in melanoma and adenocarcinoma mouse models have found that ACC1 inhibition activates the retinoic acid receptor and TGF‐β–SMAD signaling pathways, thereby promoting Th9 cell differentiation and IL‐9 production [173]. This enhances antitumor effects and significantly increases the number of tumor‐infiltrating immune cells. The differentiation and function of Th9 cells are regulated by FAM further elucidating the impact of lipid metabolic pathways on immune cell functionality. In conclusion, ACC1 plays a pivotal role in immune cell activation, differentiation, and function in the TME by regulating cellular energetics and lipid utilization, serving as a crucial regulator of tumor immune evasion and therapy resistance.

SCD is a rate‐limiting enzyme that catalyzes the conversion of saturated FAs to MUFA, of which SCD1 is the major isoform of SCD, which primarily converts stearic and palmitic acids to oleic and palmitoleic acids [174, 175]. Katoh et al. [176] found that SCD1 affects CD8+ effector T cell function, inhibits DC recruitment, and attenuates antitumor immune responses via Wnt/β‐catenin signaling and endoplasmic reticulum stress. Additionally, their research revealed that SCD1 exhibits direct immunosuppressive activity on CD8+ T cells, although the potential molecular mechanism still remain to be fully elucidated [176]. Recent investigations have established that SCD1 attenuates the effector function of antitumor CD8+ T cells through the upregulation of acetyl‑CoA acetyltransferase 1 (ACAT1)‐mediated immunosuppressive oleic acid‐associated esterified cholesterol production [177]. Collectively, these findings reveal the role of SCD1 in tumor immune escape and provide new molecular mechanisms as well as potential therapeutic strategies for cancer immunotherapy.

#### 4.2.3 FAM and Tumor Therapeutic Resistance

In recent years, FAM critically affects chemotherapy resistance in cancer treatment [178]. As cancer cells develop resistance to chemotherapeutic drugs, they frequently undergo significant alterations in their metabolic pathways—a phenomenon known as metabolic reprogramming [26, 179]. Specifically, cancer cells that develop resistance to cisplatin shift from a primarily glucose‐dependent glycolytic metabolism to a reliance on FA synthesis and metabolism for energy production [179]. This metabolic shift enables cancer cells to more efficiently utilize FAO for energy production and redox balance maintenance, thereby conferring chemoresistance through evasion of drug‐induced cytotoxicity and enhancing survival [27, 30]. Recent studies on tumor metabolic reprogramming have revealed multiple novel strategies to reverse chemotherapy resistance. Among these, the calcification‐inducing drug FpSA blocks FA uptake by downregulating the FA transporter CD36 and the binding protein FABP4 in cisplatin‐resistant cervical cancer cells [180]. The microcalcification it induces further disrupts mitochondrial function, inhibits FA β‐oxidation, and forces metabolic reversion to glycolysis, thereby effectively reversing cisplatin resistance [180]. In lipid synthesis pathways, a short peptide mimicking the Insig1/2 protein's loop 1 region specifically binds to and inhibits phosphorylated PCK1, thereby blocking SREBP‐mediated abnormal lipid synthesis [181]. Delivery of this peptide via lipid nanoparticles not only significantly suppresses HCC growth in preclinical models but also exhibits synergistic effects with the first‐line targeted drug lenvatinib [181]. Additionally, targeting very long‐chain FA (VLCFA) metabolism shows potential for therapy sensitization: inhibiting peroxisomal acyl‐CoA oxidase 1 (ACOX1) leads to VLCFA accumulation, which disrupts the interaction between key kinases MET and IGF1R and significantly enhances the efficacy of proteasome inhibitors such as bortezomib against resistant tumors [182]. In cancer therapeutics, a distinct subpopulation of drug‐tolerant cells termed “persisters” emerges as key mediators of chemoresistance [141, 183, 184]. The emerging significance of FAO in these persistent cells is attracting increasing attention, and it promotes chemoresistance and cancer progression through multiple ways. Studies have found that persistent cells in melanoma rely on peroxisomal FAO activated by the PPARα–PGC1α–ACOX1 axis to maintain their survival [185]. Cancer stem cells (CSCs) are attracting significant attention due to their crucial roles in tumor progression, metastasis, chemoresistance, and recurrence [186]. CSCs exhibit high resistance to chemotherapeutic agents, allowing them to escape drug‐induced cell death and leading to posttreatment tumor recurrence [187, 188]. Studies have revealed that CSCs, through metabolic reprogramming, specifically via JAK/STAT3‐regulated FA β‐oxidation, promote breast CSC characteristics and chemoresistance [189]. Similarly, FAO is fundamentally required for maintaining the tumorigenic capacity and chemotherapy resistance of pancreatic CSCs, and modulation of its metabolism can enhance or suppress these cell characteristics by influencing energy metabolism and cell death pathways [190]. Furthermore, mounting evidence highlights the important role of mesenchymal stem cells (MSCs) in tumor chemoresistance. Mechanistically, MSC‐secreted TGF‐β1 activates SMAD2/3 via TGF‐β receptors and induces the expression of long noncoding RNA (lncRNA) MACC1–AS1 in gastric cancer cells [191]. MACC1–AS1, in turn, promotes FAO‐dependent stemness and chemoresistance by antagonizing miR‐145‐5p [191]. Interestingly, research has also shown that MSC‐induced lncRNA HCP5 promotes FAO, thereby fostering gastric cancer stemness and chemoresistance, via the miR‐3619‐5p/AMPK/PGC1α/CEBPB axis [192]. Both CSCs and MSCs exhibit characteristics of persistent cells; however, CSCs directly contribute to tumor chemoresistance and recurrence, while MSCs exert their influence indirectly by modulating the TME. In summary, chemotherapy‐resistant tumor cells exhibit a dependence on FAO, likely due to its ability to provide additional energy and antioxidant capacity, enabling these cells to adapt to and withstand the effects of chemotherapeutic agents. Targeting metabolic pathways and signaling cascades involved in this process represents a promising approach to conquer chemoresistance in cancer therapy.

Radiotherapy, a widely employed cancer treatment modality, primarily exerts its therapeutic effects through DNA damage, particularly DNA double‐strand breaks [193, 194]. Following radiotherapy, cancer cells undergo metabolic reprogramming, increasing FAM to provide ample substrates and energy for DNA damage repair, thereby mitigating cytotoxic effects and contributing to radioresistance [194, 195, 196]. MUFA (such as oleic acid and palmitoleic acid) are significantly upregulated in radiation‐resistant cancer cells. These FAs protect cancer cells from radiation therapy damage by inhibiting ferroptosis driven by lipid peroxidation through activation of the long‐chain acyl‐CoA synthase 3 (ACSL3) protein [197]. In radioresistant cancer cells, the FAO process is particularly active, with a consistent upregulation of CPT1A, the rate‐limiting enzyme of FAO, observed in these cells [198, 199]. Specifically, Tan et al.’s analysis of radioresistant nasopharyngeal carcinoma revealed significantly enhanced FAO activity and upregulated CPT1A protein expression when compared with radiosensitive cells [198]. Furthermore, CPT1A inhibition can activate mitochondrial apoptosis and resensitize previously radioresistant cells to radiotherapy [198]. In follow‐up studies, they demonstrated that the PGC1α/CEBPB/CPT1A/FAO axis critically promotes radioresistance in nasopharyngeal carcinoma [199]. FAM‐induced immunosuppression is a key mechanism in cancer radioresistance. For example, Jiang et al. [200], in their research on glioblastoma, found that FAO provides ATP through catabolism within cellular mitochondria, helping tumor cells evade the cytotoxic effects of radiotherapy. This process promotes glioblastoma radioresistance through CD47‐mediated immune evasion [200]. Furthermore, recent studies have revealed that PITPNC1‐mediated FASN regulation of CD155 suppresses CD8+ T cell responses and enhances colorectal cancer radioresistance [201]. FASN plays a pivotal role in this process: FASN knockdown not only reduces cell proliferation and survival by inhibiting the AKT/ERK/AMPK pathway and induces apoptosis by increasing the BAX/p‐Bcl‐2 ratio, but also simultaneously suppresses cell migration and enhances radiosensitivity [202]. In summary, these findings support a close link between alterations in FAM and the development of radioresistance.

Endocrine therapy is fundamental in managing hormone‐sensitive malignancies. This therapeutic approach is particularly critical in breast and prostate cancers, where tumor cells demonstrate dependence on hormone signaling pathways for survival [203, 204]. Despite its therapeutic significance, the emergence of resistance to endocrine therapy represents a major clinical challenge, compromising its long‐term efficacy. Against this backdrop, the impact of alterations in FAM on endocrine therapy resistance has become a focal point of research. Altered FAM is associated with endocrine therapy resistance, particularly in estrogen receptor‐positive breast cancer [205]. In ER‐positive breast cancer cells resistant to tamoxifen, elevated FAO activity and upregulated CPT1 expression represent critical molecular drivers of endocrine resistance. Duan et al.’s research found that AKT and AMPK pathway activation promotes metabolic reprogramming and enhanced autophagy in tamoxifen‐resistant breast cancer cells [206]. Specifically, inhibition of AKT activity triggers a feedback mechanism activating AMPK, which in turn triggers the ERRα/PGC‐1β‐MCAD/CPT‐1 signaling axis. This pathway enhances FAO and autophagy, thereby attenuating the efficacy of endoxifen (the active metabolite of tamoxifen) and AKT inhibitors [206]. Furthermore, through bioinformatics and functional studies, scientists have found that the activation of FAO and overexpression of CPT1A are associated with c‐Jun activation and increased chromatin accessibility [207]. Targeting the JNK/c‐Jun–CPT1A–FAO signaling axis offers a potential therapeutic for overcoming resistance. Emerging evidence has also revealed that abnormally low expression of GPR81 in tamoxifen‐resistant breast cancer cells impairs Rap1 signaling, leading to upregulated PPARα and CPT1 expression, enhanced FAO, impaired lipid accumulation and LD formation, and suppressed autophagic activity [208]. These changes ultimately contribute to the proliferation of resistant cells. These findings both elucidate the complex molecular basis of tamoxifen resistance in breast cancer and provide promising therapeutic targets for intervention strategies. Androgen deprivation therapy (ADT) serves as the major therapeutic intervention for prostate cancer, inhibiting disease progression through systemic androgen suppression [209,210]. However, as treatment progresses, prostate cancer becomes resistant to ADT, resulting in the emergence of castration‐resistant prostate cancer (CRPC) [209]. A characteristic feature of CRPC is the reactivation of the androgen receptor (AR) signaling pathway. This reactivation is strongly associated with reprogramming of FAM, and AR signaling is a driving factor for prostate cancer progression and endocrine therapy resistance [211, 212]. For example, AR‐induced ACSL medium‐chain family members 1 and 3 (ACSM1 and ACSM3) mediate FAO in prostate cancer to enhance energy production [213]. This metabolic pathway utilizes medium‐chain FAs to produce energy, while also preventing ferritin accumulation and promoting the development of resistance to clinically approved antiandrogen therapies. Recent studies indicate that targeting key enzymes in FAM, as monotherapy or combined therapy, can restore sensitivity to AR‐targeted therapies and show potential for delaying and overcoming resistance to AR‐targeted therapies [210, 211, 214, 215, 216]. Specifically, inhibiting CPT1 to reduce FAO, or inhibiting FASN to decrease FA synthesis, both enhance the efficacy of AR antagonists [214, 216]. Although endocrine therapy remains a cornerstone of breast and prostate cancer treatment, the pervasive development of treatment resistance underscores the critical need for innovative therapeutic approaches and agents to improve clinical outcomes. Currently, combination therapies for endocrine therapy‐resistant patients are being actively investigated with the aim of providing more effective therapeutic choices for these individuals.

An increasing amount of research reveals the complexity of FAM pathways in drug‐resistant cancers, which encompasses both FA synthesis [217] and FAO [218]. These metabolic pathways synergistically fuel the OXPHOS system, meeting cellular energy demands and protecting cells from apoptosis. Multiple cancer types exhibit upregulated FASN expression, a central driver of FA production, which is strongly associated with resistance to targeted therapies. For example, in EGFR‐mutated NSCLC, FASN promotes palmitic acid synthesis, leading to resistance against targeted cancer treatments [219]. Similarly, in melanoma, the persistent overexpression of FASN is correlated with enhanced therapeutic resistance, and pharmacological inhibition of FASN enhances tumor cell sensitivity to ROS inducers, delaying the development of resistance and improving survival rates [220]. Of note, increased FAO may contribute to the acquisition of therapeutic resistance in mice during prolonged treatment with BRAF inhibitors (BRAFi) [221]. Researchers have demonstrated that intervening in FAO and the methionine salvage pathway using ranolazine can improve the efficacy of targeted melanoma therapies and may resensitize BRAFi‐resistant tumors to immunotherapy [221]. Notably, the HER2 signaling pathway directly upregulates FASN expression, while reciprocally, elevated FASN activity amplifies HER2‐mediated oncogenic signaling, creating a feed‐forward loop that sustains tumor cell proliferation [222]. Therefore, inhibiting FASN activity is regarded as a promising approach to surmount resistance in cancer cells to HER2‐directed therapies. For example, in HER2‐positive gastric cancer, FASN expression is markedly elevated in treatment‐resistant tissues compared with normal tissues. The FASN inhibitor TVB‐3166 has shown promise in overcoming resistance to HER2‐targeted therapies, as evidenced by both in vitro and in vivo experiments [223]. Beyond its association with FA synthesis, activation of the HER2 signaling pathway is also strongly associated with FAO. Recent research highlights the importance of targeting FAO, specifically inhibiting carnitine palmitoyltransferase 1a (Cpt1a), in conjunction with HER2‐targeted therapies [224]. This approach has shown considerable potential for overcoming resistance in patients with HER2‐positive breast cancer. In conclusion, these findings elucidate the molecular mechanisms underlying metabolic adaptation in drug‐resistant tumors and support the part of FAM in promoting resistance to targeted therapies, providing a strong rationale for developing next‐generation treatment approaches.

**FIGURE 4:**
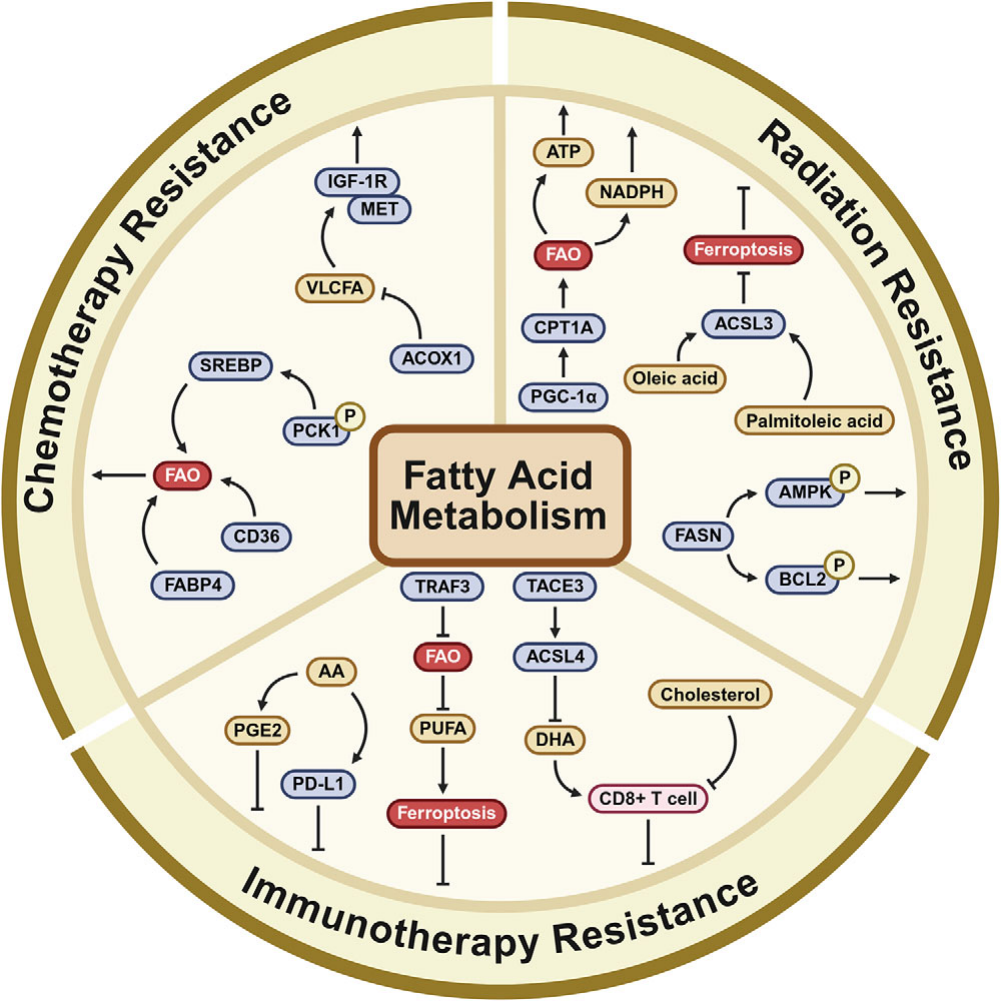
The role of fatty acid metabolism in mediating resistance to radiotherapy, chemotherapy, and immunotherapy. This figure summarizes key mechanisms by which fatty acid metabolism (FAM) contributes to therapy resistance in cancer. FAM interacts with three major forms of treatment resistance: radiotherapy, chemotherapy, and immunotherapy. The surrounding molecular and signaling pathways illustrate how altered FAM, including fatty acid uptake, synthesis, oxidation, and lipid species accumulation—modulates cell survival, ferroptosis sensitivity and immune cell function to promote treatment resistance. These pathways highlight lipid metabolism as a convergent node in multiple therapy‐resistant phenotypes.

Immunotherapy, particularly immune checkpoint blockade (e.g., anti‐PD‐1/PD‐L1 antibodies), has revolutionized cancer treatment. However, a substantial proportion of patients exhibit either innate or acquired resistance to these therapies [225, 226]. Emerging evidence highlights that FAM is a crucial mechanism underlying this resistance, primarily by forming an immunosuppressive TME that diminishes antitumor immunity [13]. The TME of many solid tumors is characterized by nutrient deprivation, hypoxia, and acidosis—conditions that drive metabolic reprogramming in both cancer and immune cells [227, 228, 229]. For example, in glioblastoma, TRAF3 deletion induces mitochondrial translocation of ECH1, which promotes PUFA β‐oxidation and thereby avoids FA peroxidation and ferroptosis caused by PUFA accumulation [230]. In metabolic dysfunction‐associated steatohepatitis‐related HCCa (MASH–HCC), squalene epoxidase and its metabolite cholesterol impair CD8^+^ T cell activity by inducing mitochondrial dysfunction while enriching Arg‐1^+^ MDSCs [231]. In triple‐negative breast cancer (TNBC), anti‐PD‐1 therapy‐resistant TNBC cells accumulate and release extracellular vesicles rich in arachidonic acid (an ω‐6 PUFA), leading to increased production of immunosuppressive molecules such as PGE2 and PD‐L1 [232]. In HCC, transforming acidic coiled‐coil protein 3 drives PUFA metabolic reprogramming in the TME by stabilizing the mRNA of ACSL4; ACSL4 is upregulated and consumes large amounts of free DHA in the microenvironment, and since DHA is a key metabolic substrate for maintaining mitochondrial function and effector capacity of CD8^+^ T cells, its depletion directly impairs the cytokine secretion and tumor‐killing ability of CD8^+^ T cells [233]. This metabolic reprogramming confers a selective advantage to immunosuppressive cell populations over effector immune cells [13]. Key immunosuppressive cells, including Tregs, TAMs, and natural killer (NK) cells, exploit FAM to sustain their survival, stability, and suppressive functions, thereby contributing to immunotherapy resistance [234]. Tregs, which normally act as “brakes” on the immune system to maintain homeostasis, can become detrimental in the TME. They suppress antitumor immune responses through various mechanisms, facilitating cancer progression [235]. Tregs exhibit remarkable metabolic adaptability, utilizing alternative metabolites such as lactate and FAs from the TME to maintain their suppressive characteristics under metabolic stress [170, 236]. The FA transporter CD36 is highly expressed in intratumoral Tregs and is believed to support Treg survival through PPAR‐β/γ‐dependent mitochondrial activity and biosynthesis. Notably, Treg‐specific knockout of CD36 significantly enhances the antitumor efficacy of PD‐1 monoclonal antibodies [166]. Similarly, PLT012, a humanized anti‐CD36 antibody, has been shown to enhance the efficacy of PD‐L1 antibodies against liver cancer [237]. Furthermore, the CD36‐targeting drug VT1021 is currently undergoing Phase 2/3 clinical trials for glioblastoma (NCT03970447) [238]. FABP5, also highly expressed in tumor‐infiltrating Tregs, directly absorbs exogenous FAs from the microenvironment and transports them to organelles such as mitochondria [239]. Knockout of FABP5 reduces the number of infiltrating Tregs in tumor and their immunosuppressive functions while significantly increasing CD8+ T cells and NK cells [240]. This suggests that FABP5 is closely linked to Treg‐mediated resistance to immunotherapy. TAMs represent the most abundant immune cell type in the TME, comprising approximately 50% of the total cell population. TAMs exhibit remarkable plasticity, differentiating into various subtypes in response to different stimuli in the microenvironment, which allows them to adapt accordingly. This high degree of heterogeneity and plasticity positions TAMs as key regulators influencing tumor progression and resistance to immunotherapy [241]. Generally, macrophages are classified into M1 and M2 types, with M1 macrophages primarily involved in antigen presentation, proinflammatory responses, and antitumor effects, while M2 macrophages are primarily responsible for suppressing inflammation and promoting angiogenesis. Therefore, M2‐like immunosuppressive TAMs are closely associated with tumor progression and resistance to immunotherapy [242]. M2‐like TAMs preferentially utilize FAO and OXPHOS for energy [243]. Therefore, their immunosuppressive functions are closely linked to lipid metabolism. For example, FABP5 expressed by TAMs facilitates the processing of unsaturated FAs derived from tumors, activating the PPARγ signaling pathway, which leads to the upregulation of immunosuppressive molecules and subsequently inhibits T cell antitumor immune responses, promoting tumor growth. Conversely, the absence of FABP5 significantly enhances T cell‐mediated antitumor activity and improves the efficacy of liver cancer immunotherapy [244, 245]. Additionally, inhibiting targets such as SREBP1, CPT1A, and CD36 can diminish the immunosuppressive phenotype of TAMs [246, 247, 248]. Collectively, these findings reveal that TAMs maintain their immunosuppressive phenotype through lipid metabolism reprogramming, which is a significant mechanism contributing to immunotherapy resistance. Interventions targeting FAM, uptake, and associated transcription factors may help modulate the immunosuppressive functions of TAMs, providing novel metabolic strategies to reverse the suppressive TME and enhance the efficacy of existing immunotherapy approaches. NK cells, as core effector cells of the innate immune system, play a critical role in tumor immune surveillance by directly killing malignant cells through the release of cytotoxic granules and proinflammatory factors, independent of major histocompatibility complex restrictions. However, lipid peroxidation‐mediated oxidative stress is a central mechanism by which the TME suppresses NK cell glucose metabolism, leading to mitochondrial damage and loss of cytotoxic function [249]. In the TME, tumor cells alter lipid metabolism, producing and releasing inhibitory lipid signaling molecules such as LysoPS. These molecules act like “metabolic immune checkpoints” by directly targeting receptors on NK cells (e.g., GPR34), activating intracellular signaling pathways such as cAMP–PKA–CREB, which suppress NK cell antitumor effects [250]. Additionally, specific FAs have been shown to impact NK cell function. Oleic acid disrupts the stability of c‐Myc protein and the activity of the P300 histone acetyltransferase, resulting in decreased histone H3K27 acetylation levels at the promoter regions of key effector genes in NK cells (such as perforin, granzyme, and interferon), thereby persistently suppressing their antitumor capabilities [251]. Interestingly, recent studies have found that lactate, another metabolite in the TME, can synergistically impair NK cell mitochondrial function and reduce NAD+ levels, thereby diminishing NK cell vitality and cytotoxic capacity from both energy metabolism and oxidative stress perspectives [228]. In summary, FAM reprogramming is a key mechanism driving resistance to tumor immunotherapy by creating a highly immunosuppressive TME that broadly undermines antitumor immune responses. Specifically, Tregs and M2‐like TAMs rely on high expression of proteins such as CD36 and FABP5, utilizing exogenous FA uptake and OXPHOS to maintain their survival, stability, and immunosuppressive functions. Therefore, targeting this metabolic network—such as inhibiting CD36 or FABP5 to relieve Treg/TAM suppression, or neutralizing inhibitory lipid signals to restore NK cell function—represents a promising new strategy for reversing immunosuppression and enhancing the efficacy of immune checkpoint blockade therapies. Future research should further explore the precise temporal and spatial regulation of different FA types and metabolic pathways on immune cells, facilitating the clinical exploration of combining metabolic‐targeted therapies with existing immunotherapeutic strategies. Overall, abnormal FAM is a key driver of multidrug resistance in tumors, profoundly influencing tumor cell responses to radiotherapy, chemotherapy, and immunotherapy through diverse mechanisms (Figure 4).

### 4.3 FAM in Other Disorders

#### 4.3.1 Diabetes

Diabetes mellitus presents a monumental and escalating global health threat. According to the World Health Organization, the number of adults living with diabetes has surged from 200 million in 1990 to 830 million in 2022, with prevalence increasing more rapidly in low‐ and middle‐income countries. This epidemic is expected to worsen, with projections indicating that the burden of early‐onset Type 2 diabetes in the Americas will continue to rise in terms of absolute deaths and disability‐adjusted life years through 2050 [252, 253]. One of the contributing factors to this concerning trend is the dysregulation of FAM, which plays a notable role in the pathogenesis of diabetes. This dysregulation establishes a complex relationship between lipid homeostasis, insulin resistance, and β‐cell dysfunction. Elevated levels of circulating FAs are closely associated with insulin resistance syndrome and increased fasting concentrations indicate an impaired ability of insulin to regulate the lipolytic pathway in adipose tissue [254, 255]. This impairment leads to an excessive influx of FFAs into peripheral tissues such as skeletal muscle and the liver, which is widely recognized as a significant contributor to metabolic dysfunction [256]. However, recent large‐scale cross‐sectional studies provide strong evidence that specific subtypes of PUFAs are linked to a reduced risk of insulin resistance. When assessed as a percentage of total circulating FAs, linoleic acid (an omega‐6 PUFA), arachidonic acid (also an omega‐6 PUFA), and DHA (an omega‐3 PUFA) are inversely associated with insulin resistance and liver steatosis. This indicates that the composition of the FA pool is crucial for maintaining metabolic health [39].

At the molecular level, the activation and routing of FAs into various metabolic pathways are regulated by specific enzyme families. The ACSL family is essential in FAM, responsible for converting long‐chain FAs into acyl‐CoA, which is a critical initial step in intracellular FAM. This step determines whether FAs are directed toward β‐oxidation for energy production or utilized for the synthesis of complex lipids [257]. Different ACSL isoforms preferentially activate FAs of varying chain lengths and degrees of saturation. For instance, ACSL4 shows a preference for PUFAs such as arachidonic acid, making it a key player in synthesizing lipid mediators with significant signaling functions and regulating ferroptosis [257, 258]. In contrast, ACSL1 preferentially activates oleic acid, which is more directly associated with energy metabolism [259]. This functional differentiation allows cells to precisely allocate the metabolic flow of FAs according to different physiological and pathological states such as energy demand, cell proliferation and stress responses, by regulating the expression of various ACSL isoforms. Similarly, the expression of different isoforms varies across tissues and localizes to distinct organelles (such as the endoplasmic reticulum, mitochondria, and LDs), determining the destination of activated FAs. For example, ACSL1, highly expressed in the liver, is localized to the outer mitochondrial membrane and endoplasmic reticulum, facilitating the guidance of FAs toward β‐oxidation [257, 260]. This characteristic links its dysfunction to metabolic diseases such as obesity, insulin resistance, and diabetic nephropathy [261, 262, 263]. Conversely, ACSL3 is enriched in the Golgi apparatus and is associated with LD biogenesis, leaning toward the use of FAs for membrane phospholipid synthesis and lipid storage [264]. Notably, ASCL5, primarily localized in the intestine, exhibits affinity for a wide range of saturated and unsaturated FAs, facilitating the activation of exogenous FAs for subsequent triglyceride synthesis and storage within cells [265, 266]. This functional differentiation enables various organs to fulfill their specific roles according to different physiological demands, allowing for the precise selection and utilization of FAs to ensure the proper execution of physiological activities.

Dysregulation of these enzymes can disrupt lipid homeostasis, contributing to the lipotoxicity observed in diabetic conditions. Under high glucose stimulation, the knockout of ACSL1 leads to approximately a 45% reduction in insulin secretion from β‐cells, indicating that ACSL1 plays a critical role in regulating insulin secretion through FAM, impacting Type 2 diabetes [267]. Ergosterol can stabilize the active conformation of ACSL1, maintaining its active closed conformation and promoting FA β‐oxidation, thereby alleviating insulin resistance [262]. Other studies have found that the knockout of ACSL3 and ACSL4 can also reduce insulin secretion from β‐cells by about 50% [268]. Additionally, the loss of ACSL5, which can absorb exogenous FAs, has been shown to increase insulin sensitivity in mice [269].

The ACSL family also plays a role in the development and progression of chronic complications associated with diabetes [269]. In the context of diabetic nephropathy, ACSL5 expression is upregulated in renal tubular epithelial cells, promoting lipid deposition and lipotoxic apoptosis, leading to tubular injury. Silencing ACSL5 can alleviate these pathological changes [270]. Similarly, the activation of ACSL1‐mediated ferroptosis is closely related to renal injury in diabetic nephropathy [261, 271]. Interestingly, lactate can also enhance the expression of ACSL4 through the regulation of epigenetics, thereby promoting ferroptosis in diabetic nephropathy cells [272]. In diabetic cardiomyopathy, ACSL4 translocates to the mitochondria in endothelial cells, inducing mitochondria‐related ferroptosis [273]. In diabetes‐related atherosclerosis, the expression of ACSL1 significantly increases in bone marrow‐derived macrophages. Conditional knockout of ACSL1 in macrophages reprograms these cells to an anti‐inflammatory phenotype, reducing inflammation in local plaques as well as systemic inflammatory responses [263].

In summary, the rising global burden of diabetes highlights the critical role of dysregulated FAM in its pathogenesis and complications. Evidence indicates that changes in the composition and flux of circulating FAs, especially the protective associations of specific PUFAs, highlight the metabolic significance of lipid species beyond their function as mere energy substrates. Central to this regulatory network is the ACSL enzyme family, which governs the metabolic routing of FAs and acts as a pivotal point influencing insulin sensitivity, β‐cell function, and systemic lipid homeostasis. The isoform‐specific localization and functional specialization of ACSLs allow for precise control over FA utilization across various tissues; however, their dysregulation is a key mechanism contributing to lipotoxicity, organ damage and inflammatory responses in diabetes.

Looking ahead, several promising avenues warrant further investigation. First, the roles of ACSL isoforms vary in different tissues. For example, ACSL1 is involved in hepatic β‐oxidation and macrophage polarization, while ACSL4 plays a role in ferroptosis‐related renal and cardiac injury. This variability suggests that selectively modulating these isoforms may offer novel therapeutic strategies. However, translating these insights into clinical applications necessitates a deeper understanding of ACSL regulation in humans. Second, while current studies emphasize the detrimental effects of ACSL overexpression in complications such as nephropathy and atherosclerosis, the potential protective or adaptive functions of certain isoforms under specific metabolic conditions remain largely unexplored. A nuanced understanding of ACSL‐mediated metabolic flexibility may not only illuminate pathophysiological mechanisms but also pave the way for personalized approaches to restore lipid metabolic homeostasis in diabetes.

#### 4.3.2 Nonalcoholic Fatty Liver

NAFLD is a metabolic stress‐related liver injury closely associated with insulin resistance and genetic susceptibility. According to the 2023 international expert consensus, the terminology has been updated to metabolic dysfunction‐associated fatty liver disease (MASLD) to more accurately reflect the metabolic nature of the disease [274]. This disease spectrum ranges from simple hepatic steatosis to MASH, which can further progress to liver fibrosis, cirrhosis, and HCC. The prevalence of NAFLD in the general adult population is approximately 30%, making it one of the most common chronic liver diseases, with rates as high as 44.37% in certain regions such as Latin America [275]. Reliable studies project that the number of NAFLD cases in the United States will exceed 100 million by 2030 [276]. Additionally, NAFLD is closely linked to metabolic disorders such as obesity, Type 2 diabetes, hyperlipidemia, and hypertension. Globally, about 75% of patients are obese, and approximately 65% have Type 2 diabetes [277, 278]. Notably, the incidence of HCC in MASLD patients is 44 per 100,000 person‐years, with some regions such as Southeast China, reaching 89 per 100,000 person‐years [279, 280]. This underscores the seriousness of NAFLD as a significant public health challenge.

The imbalance in hepatic lipid metabolism leading to hepatic steatosis is a primary characteristic of NAFLD and profoundly impacts its pathological processes. In a healthy state, the liver maintains lipid homeostasis through precise regulation of FA uptake, synthesis, oxidation, and export. However, in NAFLD, this dynamic balance is disrupted, resulting in excessive lipid accumulation in the liver [281]. Studies have identified four main pathways associated with hepatic fat accumulation in NAFLD patients: increased influx of FFAs, enhanced DNL, impaired FAO and insufficient secretion of very low‐density lipoprotein [282]. This section will focus primarily on the increased influx of FFAs. Adipose tissue serves as an energy reservoir, where triglycerides are synthesized from circulating FAs after meals. Most NAFLD patients exhibit insulin resistance, causing FFAs to escape from adipose tissue and be absorbed by the liver through alternative pathways. When the liver's capacity to manage and export triglycerides in the form of FFAs becomes overwhelmed, hepatic lipotoxicity occurs, leading to NAFLD. Research has shown that in liver samples from NAFLD patients, levels of FA transport‐related proteins CD36, FABP4, and FABP5 are abnormally elevated [283, 284]. Basic research on these FA transport proteins has demonstrated remarkable therapeutic potential. Knockout of CD36, FABP4 or FABP5 can reduce fat uptake and alleviate fatty liver in mice [285, 286, 287]. Among these, research on CD36 is particularly prominent, especially regarding its association with various posttranslational modifications (PTMs). Palmitic acid, as a type of FFAs, can mediate the palmitoylation of CD36, playing a significant role in several diseases, including myocardial infarction, breast cancer, and diabetes [288, 289, 290]. In NAFLD, palmitoylation of CD36 promotes its translocation to the cell membrane and enhances its binding and uptake of long‐chain FAs, thereby facilitating the development and progression of NAFLD [291]. The tumor suppressor gene EVA1A can improve hepatic FA uptake and β‐oxidation by reducing palmitoylation of CD36 [292]. Additionally, protocatechuic acid can inhibit the palmitoylation of CD36, reducing lipid uptake and accumulation in hepatocytes. Interestingly, multiple in vitro studies have found that treatment with palmitic acid can also significantly induce CD36 expression [293, 294]. This suggests that palmitic acid may influence both CD36 expression and PTMs at the same time. Furthermore, CD36 is regulated by other PTMs. O‐GlcNAcylation at S468 and T470 protects CD36 from ubiquitin‐dependent degradation, promoting its membrane translocation, which exacerbates hepatic steatosis and fibrosis in mice [294]. Additionally, arsenic exposure‐induced H3K18 lactylation promotes CD36 expression, activating the NLRP3 inflammasome and exacerbating lipid deposition and inflammatory responses in hepatocytes [295]. The evidence suggests that the functional abnormalities of FA transport proteins, such as CD36, are particularly critical in the lipid accumulation observed in NAFLD patients. These proteins precisely regulate their expression and activity through multiple PTMs, including palmitoylation, O‐GlcNAc modification, and epigenetic regulation, thereby driving lipid accumulation in hepatocytes. Targeting this regulatory network particularly CD36 and its modifying enzymes holds promise for developing novel therapeutic strategies aimed at restoring hepatic lipid homeostasis.

Subsequent studies have found that the triglycerides, FFAs, cholesterol, and ceramides accumulated in the livers of NAFLD patients can enter the peripheral circulation, leading to systemic metabolic stress and causing lipotoxic responses in multiple organs, resulting in mitochondrial dysfunction, endoplasmic reticulum stress, and apoptosis [39, 296, 297, 298]. This realization underscores that NAFLD is a systemic disease. NAFLD and chronic kidney disease (CKD) share numerous common risk factors and are closely related in terms of pathophysiological mechanisms. Research has shown that NAFLD is significantly associated with approximately a 1.45‐fold increased long‐term risk of developing CKD stage ≥3 [299]. Biologically, NAFLD can lead to kidney damage through various pathways, including: ① increased release of cytokines (such as IL‐6, TNF‐α, CRP, TGF‐β) [300, 301]; ② activation of the JNK and NF‐κB pathways, promoting systemic inflammation and oxidative stress [302, 303]; ③ the presence of the renin–angiotensin system (RAS) in adipose tissue and the liver [300]; ④ direct renal damage due to insulin resistance [304]. Furthermore, studies from multiple regions worldwide have indicated that NAFLD is associated with an increased incidence of various arrhythmias [305]. The potential mechanisms by which NAFLD promotes arrhythmias include: ① structural changes: NAFLD can lead to myocardial fibrosis and hypertrophy, altering the physical properties of the cardiac matrix and disrupting the normal conduction of electrical impulses in the heart, creating an anatomical basis for conditions such as atrial fibrillation [306, 307]; ② electrophysiological abnormalities: NAFLD‐related metabolic disturbances (such as insulin resistance and oxidative stress) can affect the function of ion channels (such as sodium, potassium, and calcium channels) in cardiomyocytes, leading to abnormal action potential duration and increased electrical instability in cardiomyocytes [308, 309, 310]; ③ autonomic nervous dysfunction: NAFLD patients often exhibit cardiac autonomic dysfunction, which lowers the threshold for arrhythmias, making them more likely to occur [305, 311]. Notably, NAFLD is associated with accelerated loss of skeletal muscle mass, which plays a crucial role in lipid metabolism; the intermediate products of lipid metabolism serve as signaling molecules that regulate insulin sensitivity, thereby closely linking skeletal muscle to the progression of NAFLD [312]. Research has shown that in the context of NAFLD, the liver itself can act as a “multiplier” of metabolic disorders. It does so by secreting substances such as SEPP1 and IGF‐1, which interfere with insulin signaling in skeletal muscle, leading to impaired glucose uptake and utilization [313, 314]. This creates a vicious cycle: “hepatic fat accumulation → insulin resistance → impaired muscle metabolic function → decreased muscle mass → exacerbated systemic insulin resistance → worsened liver disease.” At the same time, skeletal muscle can actively regulate the progression of NAFLD. As a muscle factor, skeletal muscle‐derived FSTL1 regulates hepatic lipid metabolism, inflammation, and fibrosis through the DIP2A/CD14 receptor pathway [315]. Additionally, skeletal muscle‐specific knockout of the KLF15 gene leads to obesity, impaired glucose tolerance, insulin resistance, and increased susceptibility to NAFLD in mice [316].

In conclusion, NAFLD/MASLD is a systemic disease initiated by hepatic lipid metabolism imbalance, influencing multiple organ systems through complex metabolic and signaling pathways. In the liver, FA transport proteins such as CD36, through palmitoylation, O‐GlcNAc glycosylation, and epigenetic regulation, become key molecular switches driving intrahepatic lipid accumulation. When lipotoxic metabolites from the liver enter circulation, they trigger insulin resistance, inflammatory responses and oxidative stress, leading to extrahepatic complications such as CKD, arrhythmias and loss of skeletal muscle through specific signaling pathways (such as cytokine release, activation of the JNK/NF‐κB pathway and actions of the RAS). Importantly, the interorgan dialogue established between the liver and skeletal muscle through factors such as SEPP1, IGF‐1, and FSTL1 forms a vicious cycle that exacerbates the overall process of metabolic dysregulation. Therefore, future research should not only continue to elucidate the intrinsic lipid regulatory networks of liver but also focus on therapeutic strategies aimed at breaking the harmful signaling exchanges between organs. Developing specific inhibitors targeting key nodes such as CD36 and its modifying enzymes, and exploring combination therapies that can simultaneously improve hepatic and extrahepatic metabolic homeostasis, will represent new directions for the prevention and treatment of NAFLD and its systemic complications.

#### 4.3.3 Atherosclerosis

Atherosclerosis is a common chronic cardiovascular disease characterized by the formation of atherosclerotic plaques composed of lipids, cholesterol, calcium, and other substances in the arterial walls. This process leads to narrowing of the vascular lumen and hardening of the vessel walls, making it a prevalent cause of heart disease and stroke [317]. Abnormal FAM plays a central role in the pathogenesis of AS. Metabolic reprogramming is evident throughout the disease, involving adaptive metabolic changes in various cell types, including endothelial cells, macrophages, and vascular smooth muscle cells (VSMCs) [318, 319]. Systematic metabolomic studies have revealed significant metabolic alterations in patients with AS, including dysregulation of branched‐chain amino acids, aromatic amino acids and their derivatives, bile acids, steroid hormones, SCFAs/ketone body intermediates (such as acetate and 3‐hydroxybutyrate), and Krebs cycle intermediates (such as citrate) [320, 321, 322]. These metabolic changes not only reflect the metabolic basis of AS but also provide important insights into the role of FAM in the progression of AS. The following sections will discuss the abnormalities in FAM in AS from the perspectives of endothelial cells, macrophages, and VSMCs.

Endothelial dysfunction is the initiating event in AS, promoting the adhesion and migration of monocytes to the lesion site through inflammatory responses, thereby playing a crucial role in the early stages of AS. Under physiological conditions, vascular endothelial cells exhibit a metabolic profile highly reliant on glycolysis rather than OXPHOS [323, 324]. This state allows cells to better resist inflammation and oxidative stress, contributing to vascular health. However, in high‐risk atherosclerotic plaques, endothelial cells show activation of proinflammatory pathways, accompanied by increased glycolysis, enhanced amino acid utilization, and reduced FAO [325, 326]. Research has found that in a subset of aortic endothelial cells with a genetic predisposition to AS, the PUFA‐metabolizing enzyme soluble epoxide hydrolase (sEH) is selectively upregulated. Endothelial‐specific overexpression of sEH accelerates the progression of AS, while endothelial‐specific knockout of sEH can resist PCSK9‐mediated plaque formation [327]. Nrf2 is a key regulatory protein in cellular antioxidant responses, and endothelial‐specific activation of Nrf2 can inhibit lipid peroxidation and inflammation, which is closely associated with improved AS [328]. Conversely, suppression of NRF2 can contribute to the onset and progression of AS by inducing ferroptosis and exacerbating oxidative stress [329]. Furthermore, pharmacological activation of NRF2 to alleviate AS has shown significant clinical potential. Compounds such as quercetin, 6‐gingerol, and polydatin can activate NRF2 to inhibit ferroptosis in endothelial cells, thereby improving AS [330, 331, 332]. Additionally, kaempferol, ginseng extract, Typhae pollen, and Nepeta angustifolia can reduce oxidative stress in endothelial cells or delay endothelial cell senescence through the Nrf2/HO‐1 antioxidant pathway [333, 334, 335, 336, 337]. This indicates that targeting metabolic reprogramming in endothelial cells and the Nrf2‐mediated antioxidant pathway may represent effective strategies to combat the development and progression of AS.

Macrophages are the most abundant component in atherosclerotic lesions, and their lipid metabolic state directly influences disease progression [338]. In the initial stages of AS, circulating monocytes are recruited to the vascular endothelium and differentiate into macrophages. These macrophages take up modified lipoproteins, such as oxidized low‐density lipoprotein (ox‐LDL), through scavenger receptors (including SRA, LOX1, SRB1, and CD36) [339]. When the cholesterol intake exceeds their clearance capacity, macrophages transform into foam cells filled with LDs, which is recognized as a hallmark and initiating event of early atherosclerotic lesions [338]. Single‐cell RNA sequencing analysis has revealed significant upregulation of phospholipase D3 (PLD3) in macrophages within atherosclerotic plaques, indicating its diagnostic value. In vitro experiments have shown that ox‐LDL can induce the expression of PLD3 in THP‐1‐derived macrophages. Mechanistically, PLD3 promotes lipid accumulation and uptake by regulating the expression of the scavenger receptor CD36, while also increasing the release of inflammatory factors such as IL‐1β and TNF‐α. PLD3 deficiency can inhibit the activation of the NF‐κB signaling pathway, highlighting PLD3's critical role in linking lipid metabolism and inflammatory responses [340]. Additionally, glycoprotein GPNMB is a key protein in macrophages for processing ox‐LDL. Targeting GPNMB with siRNA–lipid nanoparticles has been shown to reduce atherosclerotic plaque burden and improve macrophage lipid metabolic status in animal models [341]. Similarly, using ROS‐responsive nanoparticles to deliver miR‐10a to restore mitochondrial function in macrophages has also shown efficacy in AS treatment [342]. Beyond traditional lipid uptake and utilization pathways, metabolic reprogramming in macrophages also manifests as increased lipid synthesis capacity. In inflammatory environments, macrophages enhance their endogenous lipid synthesis to meet the demands of rapid proliferation and membrane assembly [343]. This process is precisely regulated by various transcription factors and enzyme systems, with FASN being a key enzyme in FA synthesis, showing significantly increased expression and activity under atherosclerotic conditions [344, 345]. Therefore, elucidating the interactive regulatory network between macrophage lipid metabolism and inflammatory responses, and developing specific intervention strategies targeting key nodes, may open new avenues for the prevention and treatment of AS.

VSMCs exhibit significant phenotypic plasticity during the progression of AS, and their metabolic characteristics undergo fundamental changes. Traditionally, foam cells were thought to primarily originate from macrophages, but recent studies have shown that over 50% of foam cells derive from VSMCs [345–347]. Research has demonstrated that the transmembrane protein TMEM41B is significantly upregulated in VSMCs derived from human atherosclerotic lesions and mouse models. By stabilizing FASN and inhibiting its ubiquitination and degradation, TMEM41B alters the intracellular lipid profile, thereby promoting FA synthesis. This TMEM41B–FASN axis not only drives lipid synthesis and enhances intracellular lipid storage but also stimulates the release of proinflammatory cytokines, contributing to the progression of AS. Interestingly, in cultured VSMCs, herpes simplex virus infection can amplify TMEM41B expression through OCT‐1‐mediated transcriptional activation, establishing a molecular link between viral infection and lipid metabolic reprogramming [345]. These findings expand the current understanding of VSMC‐derived foam cell formation and suggest that targeting the TMEM41B–FASN axis may represent a promising therapeutic strategy for AS.

In summary, the pathological processes of AS are closely related to metabolic reprogramming in various cell types in the vascular system, displaying distinct cell‐type specificity. Endothelial dysfunction is characterized by activated glycolysis and reduce FAO, with its metabolic state intertwined with the Nrf2‐mediated antioxidant pathway, collectively regulating early inflammatory responses. Macrophages uptake modified lipids extensively through scavenger receptors (such as CD36), and their lipid metabolic state, regulated by key molecules like PLD3 and GPNMB, forms a positive feedback loop with inflammatory signals such as NF‐κB, promoting foam cell formation. Importantly, VSMCs also serve as a significant source of foam cells, with their phenotypic transformation process precisely regulated by novel metabolic pathways such as the TMEM41B–FASN axis, revealing the crucial role of lipid synthesis pathways in disease progression. These findings not only elucidate the synergistic mechanisms of lipid metabolism and inflammatory responses in AS but also highlight the enormous potential for developing specific intervention strategies targeting different cellular metabolic characteristics. Future research should focus on deciphering the metabolic interaction networks among multiple cell types, advancing the treatment of AS from traditional lipid‐lowering approaches to precision regulation of metabolic immunity.

#### 4.3.4 Alzheimer's Disease

Alzheimer's disease is a progressive neurodegenerative disorder that has become an increasingly serious global health issue. By 2050, it is estimated that 152 million people worldwide will be affected by AD and other dementias [348]. Despite decades of research primarily focusing on Aβ plaques and neurofibrillary tangles, growing evidence indicates that lipid metabolism dysregulation plays a critical role in the pathogenesis of AD [349]. The brain is one of the organs richest in lipid content and diversity, and changes in FA composition due to natural aging may contribute to neurofunctional decline [350]. This section will systematically elucidate the significant role of FAM in AD.

Disruption of brain lipid homeostasis is closely linked to AD pathology, with notable abnormalities in FAM observable in the early stages of the disease [351]. Studies have shown that the accumulation of LDs appears even before the formation of neurofibrillary tangles in AD model mice [352]. This lipid metabolic disturbance is evident in both the peripheral and central nervous systems of AD patients. A study involving 287 participants demonstrated a negative correlation between fecal SCFAs and amyloid status, particularly noting reduced abundances of propionate, isobutyrate, and propionate‐producing bacteria in amyloid‐positive participants [353]. Furthermore, supplementation with propionate in drinking water reduced neuroinflammation, amyloid plaque deposition, and IL‐17 levels in AD model mice [354]. These findings suggest that FA metabolites derived from gut microbiota may modulate the pathological progression of AD through the “gut–brain axis.” This protective effect is partly attributed to the significant anti‐inflammatory actions of SCFAs, which exert their effects through receptors (such as FFAR2 and FFAR3) or epigenetic mechanisms (such as inhibiting histone deacetylases) [355–357].

Additionally, microglia, the resident immune cells of the central nervous system, play a crucial role in neuroinflammation associated with lipid metabolism dysregulation in AD. In AD, activated MG exhibit significant accumulation of LDs [358, 359]. Research has found that MG located near Aβ plaques have a higher LD load, and the intracellular LD content is negatively correlated with phagocytic activity [360]. This indicates a close relationship between LD accumulation and impaired phagocytic function in MG [361]. Subsequent studies confirmed that Aβ directly alters the lipid composition of MG and promotes LD formation. Accumulated LDs in MG within aging brains exhibit dysfunction and a proinflammatory state, characterized by defects in phagocytic function, elevated levels of ROS, and increased secretion of proinflammatory cytokines [362]. This creates a vicious cycle that exacerbates neurodegeneration. Fortunately, several potential drugs have been identified that can inhibit lipid accumulation in MG and alleviate associated functional disturbances. Succinyl phosphonate can reduce lipid accumulation and lipid peroxidation under inflammatory activation by modulating metabolic enzyme activity, which may relate to the alleviation of the aging phenotype in MG [363]. 3‐O‐cyclohexane carbonyl‐11‐keto‐β‐boswellic acid binds with high affinity to the key enzyme of FA β‐oxidation, MFE‐2, stabilizing its levels and thereby inhibiting MG overactivation, improving neuroinflammation and pathological damage in AD [364]. Interestingly, some interventions exert their anti‐inflammatory effects by promoting LD formation. For example, the triglyceride lipase inhibitor NG‐497 upregulates melatonin receptor 1A (MTNR1A) in human MG, thereby suppressing inflammation, even though it simultaneously increases LD accumulation [365]. Similarly, in a multiple sclerosis (MS) model, abscisic acid enhances the phagocytic ability of macrophages/MG to degrade myelin debris, which supports the maintenance of homeostasis in the neuroenvironment and promotes repair processes (remyelination) [366], despite also increasing the LD load in MG. This suggests that, under certain conditions, LD formation may represent a protective response of cells to stress.

In summary, lipid metabolism dysregulation plays a central role in the pathological progression of AD. Research indicates that from the neuroprotective effects of SCFAs derived from the peripheral “gut–brain axis” to the dynamic accumulation of LDs within MG, abnormalities in lipid metabolism profoundly influence neuroinflammation and cellular function. Notably, LD accumulation exhibits dual characteristics: it is associated with impaired phagocytic function and a proinflammatory phenotype, yet under specific conditions, it may serve as a protective adaptation for cells. These findings not only deepen our understanding of the metabolic mechanisms underlying AD but also provide new directions for developing precise lipid metabolism‐targeted therapeutic strategies.

#### 4.3.5 Summary and Perspectives

This section systematically elucidates the central role of FAM dysregulation in a range of significant chronic diseases, including diabetes, NAFLD/MASLD, atherosclerosis, and Alzheimer's disease. Although these conditions affect different organs and present varied clinical manifestations, they share a common pathological foundation—an imbalance in FAM. This imbalance is reflected not only in alterations to circulating FA levels and composition but also in the functionality of organelles, cellular metabolic reprogramming, and interorgan communication.

Across these diseases, certain common mechanisms of FAM dysregulation emerge. First, there is a disruption of lipid homeostasis, evident from the uncontrolled lipolysis in diabetes leading to an overflow of FFAs, to the imbalance of FA uptake, synthesis, and oxidation/output in NAFLD, and to the excessive uptake and endogenous synthesis of modified lipids by macrophages and VSMCs in AS. At the core of these processes is the breakdown of balance between lipid input, synthesis, and clearance. Second, specific metabolic reprogramming occurs in different cell types, with distinct adaptive responses to FAM dysregulation. For instance, in AS, endothelial cells favor glycolysis while reducing FAO, macrophages enhance lipid uptake and synthesis, and VSMCs drive lipid synthesis through the TMEM41B–FASN axis, transforming into foam cells. In AD, MG exhibit abnormal accumulation of LDs and functional impairment. Finally, lipotoxic derivatives and interorgan communication play critical roles: accumulated lipids and their derivatives (such as oxidized lipids) not only induce local lipotoxicity and cell death (such as ferroptosis) but also act as signaling molecules that drive harmful interorgan communication [367]. For example, in NAFLD, the liver secretes factors like SEPP1 and IGF‐1 that influence insulin sensitivity in skeletal muscle; in AD, SCFAs derived from gut microbiota regulate neuroinflammation through the “gut–brain axis.” However, it is essential to recognize the significant contextual specificity of FAM across different diseases. For example, LD accumulation in MG in AD is primarily associated with impaired phagocytic function and a proinflammatory state, whereas, under certain conditions (such as specific drug interventions), their formation may represent a protective response. Similarly, different isoforms of the ACSL enzyme family play markedly different, even opposing, roles in various tissues and diseases, highlighting the complexity of the FAM network and the necessity for precise regulatory mechanisms.

Future research should prioritize several emerging areas to translate our understanding of FAM into novel therapeutic strategies. First, developing isoform‐specific modulators targeting key metabolic enzymes, such as members of the ACSL family and FA transporters, represents a promising direction. Given the tissue‐specific functions and distinct substrate preferences of these isoforms, such precision targeting could correct pathological lipid accumulation while preserving essential metabolic functions. Second, the role of PTMs in regulating FAM merits greater attention. Research should focus on how modifications such as palmitoylation, O‐GlcNAcylation, and lactylation fine‐tune the activity and localization of metabolic regulators like CD36, and whether targeting the corresponding modifying enzymes offers therapeutic advantages over direct inhibition of the transporters themselves. Third, the spatial organization of lipid metabolism within cells presents exciting opportunities. Advanced imaging techniques and spatial omics should be employed to map the dynamic interactions between LDs, mitochondria, and other organelles in disease contexts. Understanding how contact sites, such as the “subcellular bearing” in yeast or mitochondria–LD interfaces in mammals, are regulated could reveal new mechanisms for modulating lipid flux. Finally, the dual nature of metabolic adaptations—where LDs serve both as protective reservoirs and as drivers of inflammation—requires careful dissection through single‐cell technologies and fate‐mapping approaches to determine when and how these processes become maladaptive.

Currently, our understanding of the role of FAM in disease has evolved from a simplistic view of “energy imbalance” to a more comprehensive understanding of “metabolic and signal network dysregulation.” Future breakthroughs in research will depend on continuing to explore molecular mechanisms while developing novel therapeutic strategies that can precisely reshape specific metabolic pathways, thereby opening new avenues for the prevention and treatment of these significant chronic diseases that afflict humanity.

## 5 FAM in the Inflammatory and TMEs: From “Original Sin” to New Hope

Abnormal regulation of FAM is the root cause of metabolic disorders. It not only establishes the core biological foundation for cancer initiation and progression but also exacerbates inflammatory responses and tumor advancement by driving metabolic reprogramming in cancer cells, promoting chronic inflammation formation, and reshaping the TME. Furthermore, it aids tumor cells in evading immune surveillance. This section systematically elucidates the role of FAM in inflammation and the TME. It first analyzes the mechanisms and impacts of its dysregulated expression as the “original sin” driving inflammation and tumorigenesis. Subsequently, it outlines strategic advances transforming into new therapeutic hopes across three domains: targeted therapies, dietary and lifestyle interventions, and biomarker development for personalized medicine. It highlights core technologies and research achievements targeting FAM, including small‐molecule inhibitors, gene editing, and nanomedicine delivery systems (NDDSs), demonstrating their potential and developmental directions in inflammation‐related cancer prevention and personalized medicine.

### 5.1 Dysregulation of FAM as “Original Sin”

Dysregulated FAM is considered the “original sin” of metabolic disorders, which provides an important biological basis for carcinoma development and progression by facilitating chronic inflammation and remodeling TME [94]. The core lies in the fact that abnormal FAM not only directly drives metabolic reprogramming in cancer cells, but promotes persistent inflammation and TME formation through multiple molecular mechanisms, thus providing the “soil” for cancer initiation and progression [86]. However, by delving deeper into the specific mechanisms underlying its role in inflammation and tumor development, we can develop novel therapeutic strategies, transforming this “original sin” into a beacon of hope for therapeutic. In the inflammatory microenvironment, dysregulation of FAM can result in the overproduction of inflammatory mediators, including prostaglandins and leukotrienes, which further amplify the inflammatory response, ultimately causing tissue damage and dysfunction [117]. Within the TME, aberrant activation of FAM provides tumor cells with additional energy and biosynthetic precursors, promoting tumor cell proliferation and survival [368]. Furthermore, tumor cells may modulate FAM to evade immune surveillance [369]. Altered FAM functionally impairs antitumor immunity, particularly by promoting the accumulation of Tregs and MDSCs, both of which facilitate tumor immune evasion [370, 371]. Therefore, targeting FA metabolic pathways could serve as a novel treatment approach for inflammation‐associated cancers, offering new avenues for cancer prevention and treatment.

### 5.2 Therapeutic Strategies Translated Into New Hope

#### 5.2.1 Therapeutic Targeting of FAM

Small‐molecule inhibitors have established themselves as promising candidates for targeting FAM, owing to their high selectivity, favorable bioavailability, and structural adaptability. This section presents a systematic review of small‐molecule inhibitors targeting FAM, focusing on three key mechanisms: FA uptake inhibition, synthesis suppression, and oxidation modulation. The initiation of FAM depends on cellular uptake, wherein CD36, a key transmembrane transporter protein, mediates the internalization of exogenous FAs and regulates metabolic homeostasis [372]. Inhibiting FA uptake can effectively reduce intracellular FA levels, thereby affecting tumor cell growth and metabolism [368]. The CD36‐targeted small molecule inhibitor, sulfo‐N‐succinimidyl oleate (SSO), inhibits CD36 function by irreversibly binding to lysine residue 164, thereby reducing FA uptake and intracellular lipid accumulation [373]. In a mouse oral squamous cell carcinoma (OSCC) model, SSO significantly inhibited OSCC cell proliferation and enhanced antitumor immune responses [374]. Furthermore, SSO also significantly inhibits the proliferation and migration of CAFs, showing good antitumor effects in HCC as well [375]. SSO has also shown therapeutic potential in inflammatory diseases, improving the inflammatory response [376]. In HIF‐2α knockout mouse models, SSO significantly suppressed the hyperactivation of NKT cells through CD36 blockade, leading to decreased secretion of IFN‐γ and IL‐4 [377]. This effectively improved the pathological characteristics of kidney ischemia–reperfusion injury and significantly alleviated the inflammatory response. Furthermore, SSO plays a positive role in spinal cord injury recovery by enhancing microglial lipophagy, promoting LD degradation, and improving inflammation [378]. FA synthesis is a core process in cellular FAM, primarily catalyzed by FASN [379]. FASN demonstrates widespread upregulation across multiple cancer types and exhibits strong correlations with tumorigenesis, disease progression, and therapeutic resistance, thereby becoming a critical target for anticancer therapeutics [380]. Over the past few years, numerous small molecule inhibitors against FASN have emerged, including orlistat, TVB‐2640, TVB‐3166, and cerulenin [381, 382]. Among these, orlistat, a classic FASN inhibitor, has shown improvements in patients with NAFLD by reducing hepatic fat accumulation, improving liver function, and significantly lowering markers of hepatic inflammation and fibrosis [383, 384]. In lung adenocarcinoma cell models, the FASN inhibitor AZ12756122 exhibits significant antitumor activity through a multitarget mechanism involving the induction of apoptosis, suppression of FA synthesis, disruption of the EGFR and Akt/mTOR signaling pathways, and reduction of cancer stem‐like cells [385]. Moreover, synergistic effects are observed when combined with EGFR–TKIs, enhancing therapeutic efficacy [385]. TVB‐3166 is a novel FASN inhibitor that has shown promising therapeutic potential in various tumor models and inflammatory diseases. In the field of oncology, Castagnoli et al. [223] demonstrated that FASN is highly upregulated in HER2‐positive gastric cancer cells. Treatment with the FASN inhibitor TVB3166 reduced gastric CSCs, suggesting that a combined targeting strategy against both HER2 and FASN may improve the efficacy of anti‐HER2 therapy. In inflammatory diseases, TVB‐3166 has demonstrated promising therapeutic effects in nonalcoholic steatohepatitis (NASH) models. By inhibiting FASN activity, it reduces hepatic lipid accumulation, lowers inflammatory cytokine expression (e.g., TNF‐α and IL‐6), and improves liver fibrosis [386]. Furthermore, recent research has indicated that cerulenin, by targeting the FASN/APP axis, impairs liver CSC characteristics. Synergistic effects with sorafenib, a targeted therapy for advanced HCC, have also been observed, enhancing therapeutic efficacy [387]. These findings suggest that FASN inhibitors hold significant promise in cancer therapy, and that combining them with other targeted therapies may further enhance treatment outcomes. While FASN is a well‐known target, ACC also plays a central role in FA synthesis as a rate‐limiting enzyme, and ACC inhibitors are advancing [388]. For example, ND‐630, as a highly effective ACC inhibitor, has shown remarkable therapeutic effects in NAFLD and NASH models [389]. Studies indicate that ND‐630 effectively decreases hepatic fat accumulation, enhances insulin sensitivity, and significantly lowers the expression of liver fibrosis markers, offering a novel therapeutic strategy for metabolic liver diseases [389, 390]. Furthermore, PF‐05221304, a novel ACC inhibitor, further expanded the clinical application prospect of ACC inhibitors and significantly improved hepatic steatosis and inflammatory response in NASH patients by suppressing FA synthesis and promoting FAO [391]. In NSCLC, ACC1 overexpression is linked to poor prognosis, and decreased DNA methylation levels may be a potential mechanism underlying this overexpression [392]. ND‐646, a powerful ACC inhibitor, blocks FA synthesis by preventing dimerization of the ACC subunit. It also blocks the activity of ACC1 and ACC2, which in turn inhibits FA synthesis and thus inhibits NSCLC tumor growth [392, 393]. Furthermore, ND‐654, a novel liver‐specific ACC inhibitor, mimics AMPK‐mediated ACC phosphorylation to inhibit hepatic DNL and HCC development [394]. Multiple studies have found that ACC inhibitors, when combined with other medications, exhibit better antitumor efficacy than monotherapy [394, 395]. It should be noted that ND‐646 and ND‐654 are currently in preclinical stages of development and have not yet entered human clinical trials. While currently in the preclinical stage, these drugs have exhibited significant antitumor efficacy, with anticipated progression to clinical trials to further assess their therapeutic potential in oncology. FAO represents a crucial cellular energy metabolism pathway, especially under energy stress conditions where cells upregulate this process to maintain energy supply. Targeted inhibition of FAO disrupts tumor cell energy homeostasis, effectively inhibiting their growth and survival [30]. CPT1, the key regulatory enzyme in FAO, exhibits elevated expression across diverse tumor types and facilitates tumor progression, establishing its significance as a therapeutic target [28]. Etomoxir is a classical CPT1 inhibitor that induces an energetic crisis in tumor cells through irreversible inhibition of the CPT1 blocking FAO and shows antitumor activity in models of glioblastoma and bladder cancer [200, 396]. However, in glioblastoma, the mechanism of action of the CPT1 inhibitor perhexiline is not through inhibition of FAO, and its antitumor activity is mediated mainly through inhibition of FYN proteins [397]. Recently, a research team isolated a fucoxanthin‐like compound 2,6‐dihydroxypeperomine B from the plant Peperomia dindygulensis and found that it can inhibit CPT1A activity and block the FAO process, resulting in the cancer cells’ inability to utilize FAs as an energetic resource, thus weakening their energy metabolism to exert antitumor effects, especially against FAO‐dependent tumors (e.g., colorectal cancer) [398]. Despite this, CPT1 inhibitors may cause side effects such as cardiotoxicity, limiting their clinical application. Therefore, further optimization of CPT1 inhibitors to improve their selectivity and safety remains an important area of current research. In conclusion, small molecule inhibitors against FAM show great potential for cancer and inflammatory disease therapeutics, modulating FAM at multiple levels and providing innovative approaches in these disorders.

In recent years, a number of small‐molecule inhibitors have advanced to the clinical trial stage, showing good application prospects, the following are the drugs currently in the clinical trial stage and their progress (Table 2). With the accumulation of more clinical data, these drugs are promising to be important clinical treatment tools in the future, offering more hope for patients. However, the clinical application of these drugs still faces many challenges, such as insufficient tissue specificity, potential side effects, and drug resistance. Future research needs to further optimize drug design to improve selectivity and efficacy.

**TABLE 2:** Therapeutic agents targeting key enzymes in FAM: Current progress in clinical trials for inflammatory diseases and cancer.

Drug	Target	Conditions	Phrase	Status	NCT number
TVB‐2640	FASN	Solid malignant tumor	I	Completed	NCT02223247
		Astrocytoma	II	Completed	NCT03032484
		Advanced breast carcinoma	II	Active	NCT03179904
		MASLD, MASH	II	Completed	NCT03938246
		KRAS mutant NSCLC	II	Active	NCT03808558
		NAFLD	II	Completed	NCT04906421
		Recurrent glioblastoma	III	Active	NCT05118776
		NASH	I	Completed	NCT05835180
		mCRPC	I	Recruiting	NCT05743621
Denifanstat/TVB‐2640	FASN	MASLD, MASH	III	Not yet recruiting	NCT06692283
		MASH	III	Not yet recruiting	NCT06594523
Aramchol	SCD1	NAFLD, NASH	II	Completed	NCT01094158
		NAFLD, HIV	II	Completed	NCT02684591
		PSC	II	Withdrawn	NCT06095986
		NASH	III	Suspended	NCT04104321

***Data sources*: ClinicalTrials.gov**.

***Abbreviations*: mCRPC: metastatic castration‐resistant prostate cancer; PSC: primary sclerosing cholangitis**.

Short‐hairpin RNA (shRNA)‐mediated interference is an effective gene silencing technology that can be utilized to investigate gene function or as a therapeutic modality by specifically reducing the target gene's expression. In diffuse large B‐cell lymphoma, shRNA‐mediated knockdown of the FASN gene, together with knockdown of genes related to the HIF‐1α or MAPK signaling pathways, effectively modulates the expression of genes involved in FAM, thereby impacting FA synthesis in tumor cells [399]. SKA3 and HIF‐1α are recognized as potential oncogenes [400, 401]. Specifically, SKA3 promotes cholangiocarcinoma (CCA) progression and chemoresistance by regulating the PARP‐dependent deubiquitination of HIF‐1α, thereby increasing FA synthesis [401]. Therefore, gene therapy strategies targeting SKA3 hold promise for enhancing therapeutic efficacy and improving prognosis in CCA patients. Recent research has revealed a significant reduction in FASN palmitoylation levels following ZDHHC20 knockout [402]. This suggests that FASN may be a major palmitoylation substrate of ZDHHC20 and that ZDHHC20 knockout significantly impacts FA metabolic pathways. Furthermore, FABP5 interacts with FASN and regulates it through the ubiquitin‐proteasome pathway; FABP5 knockdown results in increased FASN expression, while FABP5 overexpression induces FASN reduction [403]. CRISPR/Cas9‐mediated FASN knockout cell models demonstrate that FASN deficiency enhances T cell‐mediated killing, impacts mitochondrial function and apoptotic pathways, and may reduce the immune escape capacity of tumor cells [171]. FASN is not only a potential immunotherapeutic target, but its inhibition may also help overcome tumor immune resistance and optimize current immunotherapy strategies. Tregs are a type of T cells with immunosuppressive functions that limit tissue damage caused by autoimmunity but also facilitate tumor cell evasion of immune surveillance. Studies have found that tumor‐infiltrating Tregs expressing CD36 may represent a novel strategy for specifically targeting intratumoral Tregs [404]. Wang et al. [166] employed gene editing techniques to ablate the CD36 gene in Tregs, which resulted in a reduction in intratumoral Tregs while concomitantly increasing the number of cytotoxic T cells, thus inhibiting tumor growth without disrupting systemic immune homeostasis. Importantly, combined targeting of CD36 and PD‐1 demonstrates synergistic antitumor effects, which significantly enhance therapeutic efficacy [166]. Furthermore, in CD8+ T cells, knockout of the CD36 gene enhanced the production of cytotoxic cytokines, thereby augmenting their efficiency in eliminating tumor cells and demonstrating a more potent antitumor effect when used together with anti‐PD‐1 antibodies [167]. In breast cancer, CD36 mediates the uptake of FAs from adipocytes to malignant cells, activating signaling pathway that promote tumor progression. Genetic attenuation of CD36 alleviates adipocyte‐induced EMT and stemness, inhibiting the invasive and metastatic potential of breast cancer cells [405]. Recent studies have shown that suppressing CD36 activity using siRNA technology weakens the proliferation and migration capacity of CAFs, elucidating a crucial role for CD36 in regulating CAF biology [375]. In conclusion, CD36 knockout represents a promising strategy for cancer therapy. Additionally, site‐specific gene knockout can modulate FA β‐oxidation pathways, resulting in tumor progression suppression. For example, Guo et al. [406] demonstrated significant inhibition of HCC proliferation and migration through hepatocyte‐specific Igκ gene knockout, which disrupted FA β‐oxidation and induced lipid storage. These findings substantiate the potential of gene therapy strategies for therapeutic manipulation of FAM in oncological contexts. Gene editing technologies can regulate the expression of genes related to FAM, thereby influencing tumor cell growth and survival.

**FIGURE 5:**
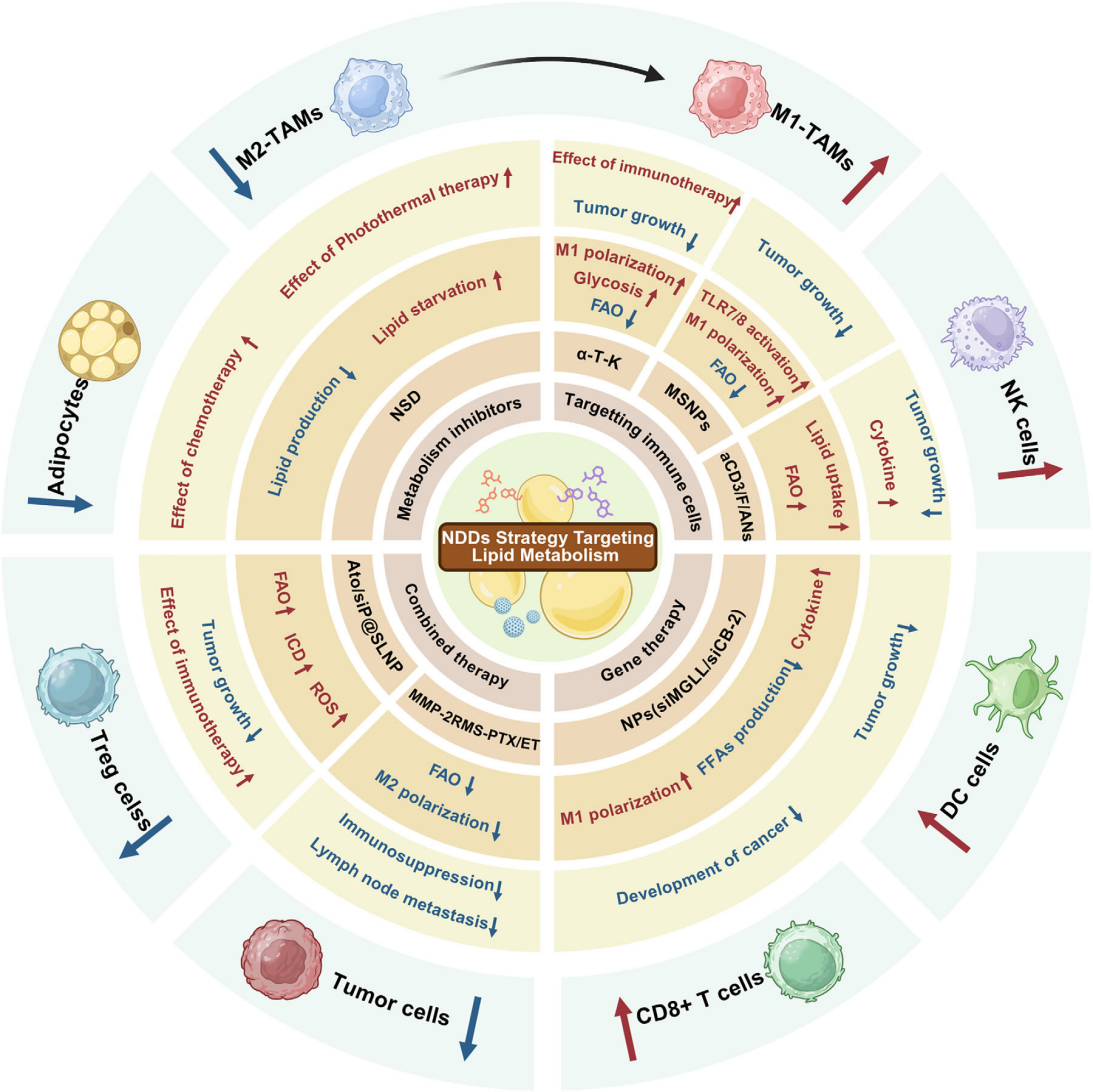
NDDS targeting fatty acid metabolism in TME. This figure illustrates a novel drug delivery strategy targeting fatty acid metabolism for cancer therapy. The strategy encompasses four key approaches: (1) metabolic inhibitors targeting key enzymes in fatty acid synthesis or oxidation; (2) immune cell‐targeted therapies modulating immune responses within the TME; (3) gene therapy approaches modifying expression of genes involved in lipid metabolism; and (4) combination therapies integrating multiple approaches.

NDDSs are carrier systems designed and constructed using nanotechnology to deliver therapeutic agents, genes, or other therapeutic molecules efficiently and precisely to target tissues or cells [407, 408]. Through site‐specific drug delivery, gene silencing, microenvironment remodeling, and combination therapies, NDDSs not only enhance drug efficacy and bioavailability but also significantly reduce adverse effects. Strategies for NDDSs targeting FAM in the TME include delivery of FAM inhibitors, targeting immune cells, combination therapy (in conjunction with chemotherapeutic agents or immunotherapy), and gene delivery systems (Table 3 and Figure 5). These strategies utilize nanotechnology to intervene in FAM from multiple perspectives, achieving precise targeting and multidimensional modulation of the TME [409, 410]. This not only directly inhibits tumor cells but also reshapes the immune microenvironment and enhances the effect of immunotherapy. With the continued advancement of nanotechnology, NDDSs will play a significant role in personalized medicine, precision therapy, and diagnosis and treatment integration, offering limitless possibilities for the future of medicine.

**TABLE 3:** List the strategies for NDDSs targeting FAM in TME.

Strategy	NDDS	Function	Targeted cell type	Condition type	References
Fatty acid metabolism inhibitor delivery	NSD	Inhibition of ACLY, synergistic treatment with lipid starvation, chemotherapy, and photothermal therapy	Tumor cell	OSCC	[411]
Targeting immune cells	α‐T‐K	Increase glycolysis and inhibit FAO, reprogram M2 macrophages	M2‐TAMs	TNBC, lung cancer	[412]
	MSNPs	Agonism of TLR7/8, inhibition of FAO, reprogramming of macrophages	M2‐TAMs	TNBC	[413]
	aCD3/F/ANs	Increase lipid uptake, activate FAO	CD8+T cells	Melanoma	[414]
Combination therapy (with chemotherapeutic agents or immunotherapy)	Ato/siP@SLNP	Promote FAO and ROS production, enhance the antitumor efficacy of PD‐L1 silencing	Tumor cell	Melanoma, colorectal cancer	[415]
	MMP‐2RMS‐PTX/ET	Inhibit FAO, block M2 macrophage polarization	Tumor cell and M2‐TAMs	Breast cancer	[416]
Gene delivery system	NPs (siMGLL/siCB‐2)	Inhibition of MGLL and CB2, along with suppression of FFAs, repolarize TAMs	Tumor cell and TAMs	Pancreatic cancer	[417]

*Abbreviation*: NSD: graphene‐based lipid modulation nanoplatform.

#### 5.2.2 Dietary and Lifestyle Interventions

Numerous research has shown that omega‐3 FAs reduce cancer risk and inhibit cancer cell growth via multiple mechanisms, including suppression of cell proliferation, induction of apoptosis, reduction of inflammation and angiogenesis, and regulation of cancer‐related gene expression [418–423]. Furthermore, the administration of omega‐3 FAs during cancer treatment can enhance the effectiveness of chemotherapy, mitigate adverse effects potentially caused by chemotherapy/radiotherapy or the cancer itself, and attenuate chemoresistance, ultimately improving patients’ quality of life [424–427]. A combinatorial therapeutic approach simultaneously targeting multiple mechanisms holds promise for enhancing the efficiency of anticancer medications and reducing risk of developing resistance [428]. However, it should be noted that the adjuvant therapeutic effect of omega‐3 FAs in radiotherapy remains inconclusive, necessitating further rigorous studies.

Saturated FAs, to some extent, induce inflammation by activating inflammatory pathways similar to those triggered by lipopolysaccharide [429], and inflammation serves as a fundamental pathogenic driver underlying numerous chronic disease states, such as cancer. In contrast to saturated FAs, unsaturated FAs, particularly omega‐3 PUFAs, generally exert the opposite effect, reducing inflammation and potentially improving cancer treatment efficacy and prognosis [429, 430]. Therefore, by adjusting dietary structure and implementing nutritional interventions targeting metabolic changes, such as reducing saturated fat intake and enhancing omega‐3 PUFA intake, FAM can be effectively regulated, inflammatory responses alleviated, and the risk of developing certain types of cancer potentially reduced [431–433].

#### 5.2.3 Biomarker Development and Personalized Medicine

Cancer patients frequently exhibit aberrant dysregulation of FAM. Precise monitoring and identification of specific biomarkers associated with FA metabolic disorders can be used in early disease diagnosis, prognosis assessment, therapeutic response evaluation, and the development of personalized treatment strategies.

The levels and activity of enzymes implicated in FAM are tightly linked to tumorigenesis, progression, and metastasis, thereby functioning as important biomarkers for predicting tumor aggressiveness and therapeutic response. Aberrant expression of critical enzymes for FA synthesis (e.g., FASN, ACC, and ACLY) is strongly linked to poor prognosis, rapid disease progression, and reduced survival in various cancers, and thus has great potential as a prognostic biomarker [434–437]. Furthermore, the ACSL family, frequently dysregulated in tumors, modulates FAM to influence multiple oncogenic processes—including endoplasmic reticulum stress, ferroptosis, drug resistance, and inflammatory microenvironment remodeling—positioning these enzymes as promising dual‐purpose biomarkers and therapeutic targets in oncology [438–443]. ACSL family members include ACSL1, ACSL3, ACSL4, ACSL5, and ACSL6 [444]. Specifically, ACSL1 shows high expression levels in ovarian cancer and lung adenocarcinoma [445, 446], while low expression of ACSL1 is correlated with poor prognosis in renal cell carcinoma [447]. ACSL3, an androgen‐responsive gene, exhibits increased expression in melanoma and NSCLC, correlating with poor prognosis in patients with these malignancies [444, 448]. In contrast, elevated levels of ACSL3 expression are linked to a more favorable prognosis in ovarian cancer [444]. ACSL4 is highly expressed in estrogen receptor‐negative breast cancer [449], while ACSL5 is upregulated in human malignant gliomas but exhibits reduced expression in colon cancer [450, 451]. In HCC, high expression of ACSL6 correlates with an unfavorable prognosis [452]. Conversely, in TNBC, high ACSL6 expression correlates with improved prognosis [453]. This discrepancy could be due to a multitude of factors, such as diverse aspects of tumor biology, heterogeneity in the TME, patient lifestyle factors, and genetic predisposition. Therefore, research focusing on the specific expression and activity of enzymes related to FAM can help to develop novel prognostic biomarkers and therapeutic targets, thereby advancing personalized medicine.

FAM‐related genes (FMGs) represent an emerging field of study as prognostic biomarkers in cancer, with an increasing amount of evidence demonstrating their significant association with cancer prognosis. Furthermore, FMGs provide a scientific basis for personalized cancer therapy and offer theoretical support and practical guidance for future drug development and treatment strategies [454–459]. For example, Huang et al. [458], through comprehensive analysis of the differential expression of FMGs, developed a prognostic model that not only predicts survival rates in colorectal cancer patients but also potentially anticipates their response to immunotherapy. Moreover, a recent study established a multifactor prognostic model based on FMGs, which was rigorously validated and found to maintain a high level of accuracy and reliability across diverse datasets and liver cancer patient populations, further substantiating the utility of FMGs as tools for evaluating cancer prognosis, improving predictive accuracy, and providing comprehensive guidance for personalized treatment strategies [459].

SCFAs, major end‐products of gut microbiota metabolism, play multifaceted roles in cancer prevention and treatment by targeting mutated genes and dysregulated pathways in tumors, promoting tumor suppressor expression, modulating energy metabolism and signaling pathways, and influencing gene expression in the TME [460–462]. Reports suggest that a microbiota metabolome centered on SCFAs could potentially act as a novel biomarker for predicting gastrointestinal cancer treatment efficacy, opening new avenues for personalized medicine and potentially constituting a therapeutic target for improving responses to ICIs [463–465]. This approach provides a novel perspective on personalized immunotherapy for gastrointestinal cancers and facilitates optimization of treatment selection for patients. However, it is worth mentioning that the investigation of SCFAs as biomarkers is still ongoing, with their role varying across different types of cancers, necessitating more scientific studies to clarify their clinical utility. For instance, recent studies have failed to directly establish SCFAs as predictive biomarkers for NSCLC, but their roles within the TME and their association with immune responses suggest that SCFAs possess considerable potential as future biomarkers for predicting immunotherapy response and monitoring tumor progression, an avenue requiring further investigation [466].

Developing personalized treatment strategies based on individual FA metabolic profiles represents a cutting‐edge and complex undertaking, requiring in‐depth analysis and comprehensive understanding of specific FA metabolic signatures in tumor tissues. By meticulously analyzing the FA metabolic profiles of tumors and integrating multiomics data, therapeutic regimens tailored to the specific metabolic characteristics of patients can be developed to predict patient response to specific metabolic modulation therapies and optimize individual cancer patient treatment [459, 467]. For example, Li et al. [467] recently developed a molecular classification method for HCC focusing on heterogeneity within the FA degradation (FAD) pathway. Through in‐depth analysis of the FAD pathway, they characterized this classification system in detail and successfully determined three subtypes of FAD with distinct clinical and biological features [467]. Personalized treatment protocols based on individual FA metabolic profiles are emerging as a promising paradigm in tumor treatment, which is expected to enhance therapeutic efficacy, minimize adverse effects, and provide patients with more precise treatment options. In conclusion, these findings collectively demonstrate the critical significance of developing FAM biomarkers to enhance the accuracy of disease diagnosis, guiding personalized treatment approaches, assessing prognosis, and monitoring therapeutic response.

## 6 Conclusion and Prospects

As a core network for cellular energy supply, structural maintenance, and signal transduction, FAM plays a dual role in maintaining physiological homeostasis and in the onset and progression of diseases, a fact that has become increasingly clear. This review systematically describes the multifunctional roles of FAM in health, as well as the mechanisms underlying its dysregulation in cancer and other chronic diseases. From fundamental enzymatic regulation to microenvironmental remodeling, from immune‐metabolic reprogramming to therapeutic resistance, abnormalities in FAM have emerged as the “common pathological basis” linking multiple chronic diseases. However, despite the considerable promise demonstrated by small‐molecule inhibitors, gene therapies, and nanoparticle delivery strategies targeting this metabolic network, their clinical translation still faces significant challenges. Among these, metabolic heterogeneity represents the core bottleneck constraining the efficacy and precision of current therapeutic approaches.

FAM exhibits significant heterogeneity across different diseases, individuals, and even among distinct cell populations within the same tumor. This heterogeneity manifests not only in the overall activity of metabolic pathways but, more critically, in the selective expression and function of key enzyme isoforms. In the field of oncology, the ACSL family and its distinct subtypes (ACSL1, ACSL3, ACSL4, ACSL5, ACSL6) exhibit differences in tissue distribution, substrate preference, and cellular localization, leading them to play diverse—and even opposing—roles across various cancers. In lung cancer and ovarian cancer, high expression of ACSL1 correlates with poor prognosis, whereas its low expression in renal cell carcinoma predicts worse outcomes. ACSL3 promotes tumor progression in melanoma and NSCLC, but may be associated with favorable prognosis in ovarian cancer. This cancer‐specific and even subtype‐specific expression pattern suggests that broadly targeting “inhibition of FAM” may yield limited efficacy and potentially induce off‐target toxicity. Beyond the ACSL family, the expression of key molecules such as CD36, FASN, and SCD1 also exhibits significant variations across tumor types and cellular subpopulations. This leads to markedly different therapeutic responses to identical metabolic interventions among patients, highlighting the formidable challenge posed by metabolic heterogeneity to precision medicine. Future therapeutic strategies must evolve toward “precision targeting,” which involves selecting inhibitors specific to particular ACSL subtypes or other metabolic nodes based on precise metabolic subtyping. This necessitates moving beyond histological classification to establish a tumor molecular subtyping system grounded in metabolic characteristics.

During treatment, monitoring changes in tumor metabolic pathway activity through dynamic PET imaging serves as a biomarker for early efficacy assessment, offering significantly greater sensitivity and predictive value compared with traditional imaging‐based evaluations of tumor size changes. Advancements in metabolic imaging technologies have provided critical tools for predicting therapeutic outcomes guided by metabolic profiling, with FAM‐specific PET imaging demonstrating broad application potential. Traditional PET imaging primarily relies on glucose metabolism probes (such as ^1^ ^8^F‐FDG), but it cannot accurately reflect the abnormal characteristics of FAM. The development and application of novel FA metabolic probes enable dynamic tracking of FA uptake, synthesis, and oxidation processes. For instance, based on the CD36‐mediated FA uptake mechanism, the development of ^1^ ^8^F‐labeled long‐chain FA probes (e.g., ^1^ ^8^F‐FTHA) enables direct visualization of tumor cell uptake activity for FFAs, facilitating the screening of CD36‐high‐expression populations suitable for targeted therapy. For FA synthesis pathways, designing PET probes labeled with acetyl‐CoA or malonyl‐CoA precursors can assess FASN and ACC activity, predicting the efficacy of related inhibitors. Probes based on the β‐oxidation pathway can distinguish FAO‐dependent tumors, guiding the clinical application of CPT1 inhibitors. Therefore, integrating metabolic imaging technology with metabolic profiling and targeted therapy to establish a closed‐loop system of “image‐guided precision metabolic medicine” represents a critical frontier for overcoming current treatment bottlenecks and optimizing clinical decision‐making.

In summary, FAM research has evolved from a classical field of bioenergetics into a core interdisciplinary discipline linking cellular fate, immune regulation, microenvironment remodeling, and disease treatment. The broad landscape outlined in this review—spanning fundamental mechanisms to therapeutic applications—highlights the immense translational potential of this field. However, the path to successful clinical translation must overcome the formidable challenge of metabolic heterogeneity. Future breakthroughs will depend on precise subtyping through multiomics integration, the development of subtype‐specific targeted therapies, and the widespread adoption of advanced metabolic imaging technologies capable of enabling personalized efficacy prediction and monitoring. By elevating our research perspective from “blocking pathways” to “remodeling networks” and “precision interventions,” we can truly harness FAM—once considered the “original sin”—and transform it into a “new hope” for curing numerous human diseases.

## Author Contributions

N. Hang, R. Zhao, and F. Zhang analyzed data and wrote the paper. D. Guo, Q. Li, Z. Shen, R. Gao, C. Gao, Z. Xie, and S. Fu helped analyze data. P. Luo, B. Tang, and L. Wang revised the paper and supervised the project. All authors read and approved the final manuscript.

## Funding

This study was supported by the 2023 Wu Jieping Foundation (Nos. 320.6750.2023‐03‐54).

## Ethics Statement

The authors have nothing to report.

## Conflicts of Interest

The authors declare no conflicts of interest.

## Data Availability

Data sharing not applicable to this article as no datasets were generated or analyzed during the current study.
